# Supplementary material for: An optogenetic toolkit for robust activation of FGF, BMP, & Nodal signaling in zebrafish
Source: bioRxiv. 2025 Apr 19:2025.04.17.649426. Preprint. [Version 1] doi: 10.1101/2025.04.17.649426 (PMC12190770; doi:10.1101/2025.04.17.649426)
Supplement: Supplement 1 [file media-1.zip › Supplementary_Materials/Supplementary_Figures.pdf]

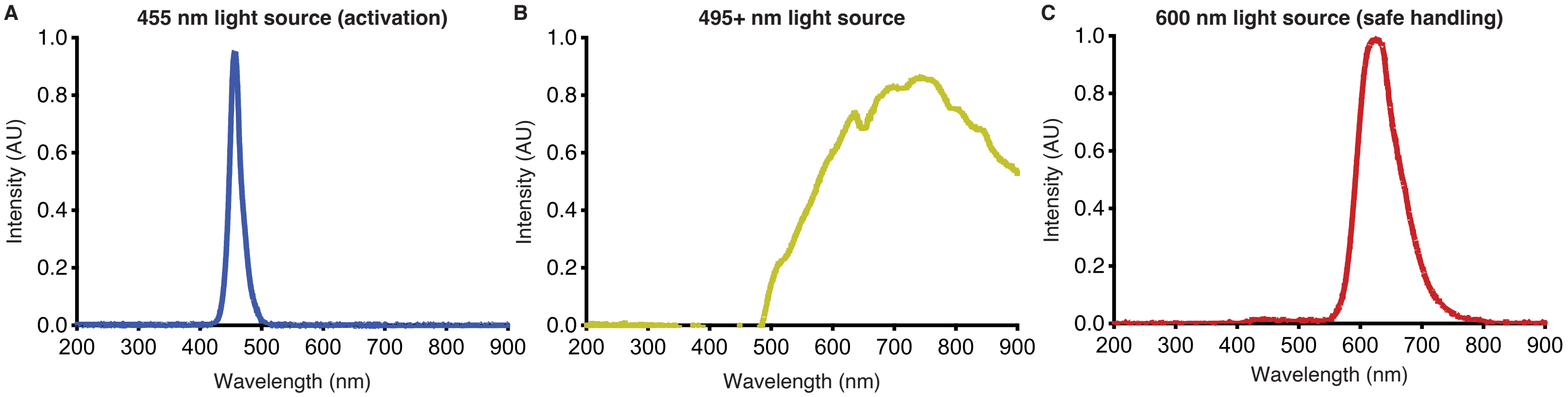

Supplementary Figure 1: Light source spectra. Measured spectra for **A)** the 455 nm light used in uniform blue light exposure experiments (all figures except Fig. 7), **B)** the 495+ nm used in Figs. 2 and Supp. Figs. 2, 3, 4, & 12, and **C)** the 600 nm light source used to avoid inadvertent signaling activation during handling of embryos expressing bOpto constructs.

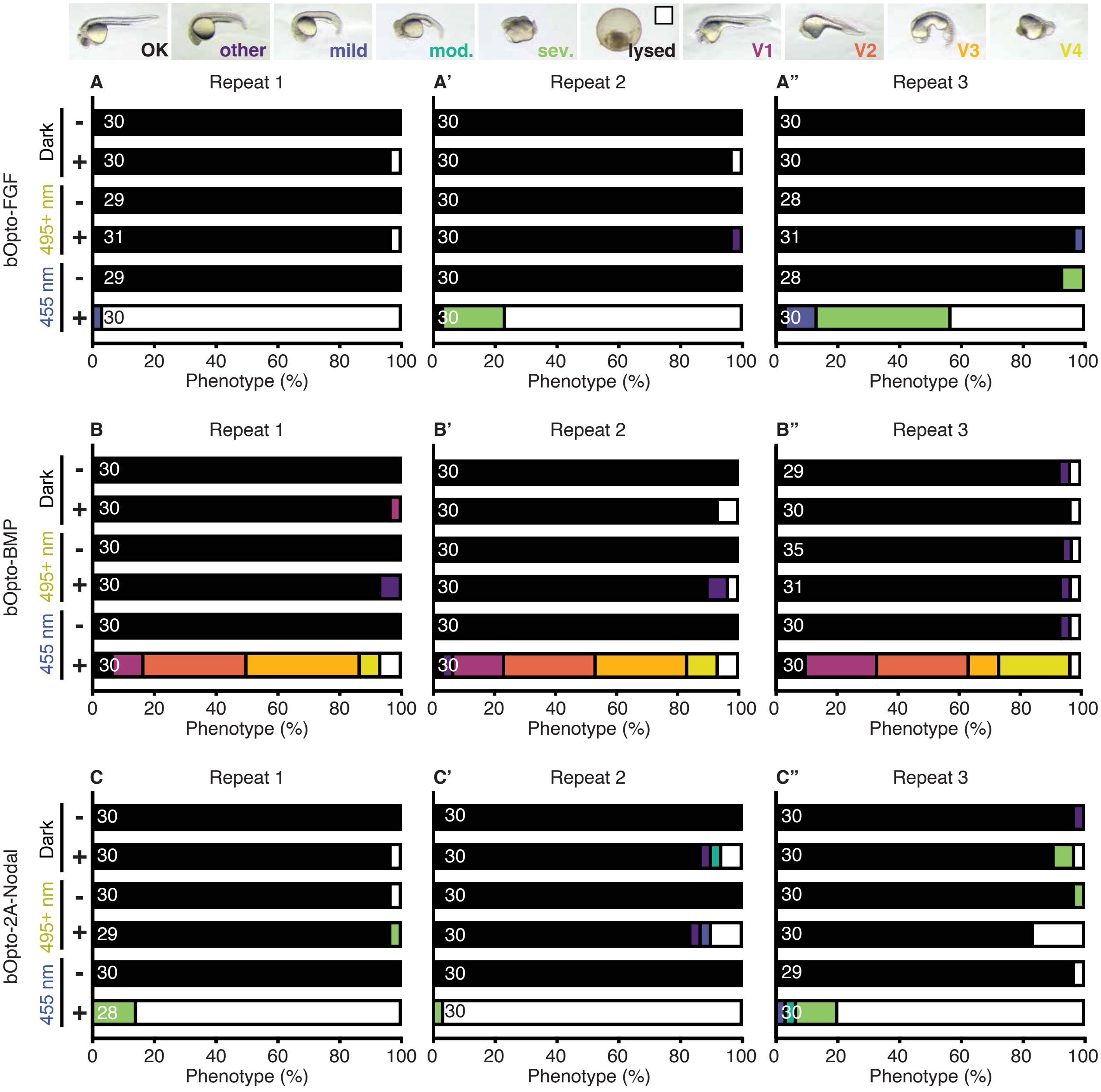

Supplementary Figure 2: Wavelength-dependent activation of FGF, BMP, & Nodal signaling experiment repeats. A-C") Uninjected embryos (-) and embryos injected (+) at the one-cell stage with the *bOpto-FGF* (A-A"), *bOpto-BMP* (B-B"), or *bOpto-2A-Nodal* (C-C") mRNA were exposed to dark, 495+ nm light (18.51 W/m^2), or 455 nm light (50 W/m^2) starting 1.5-2 hours post-fertilization. Phenotypes were scored at 1 day post-fertilization. Individual repeats are shown. Numbers indicate the total number of embryos scored in each condition for that experiment. Figure 2A shows the combined data from these three repeats. Phenotype legend images shown here are the same images shown in Figure 2A.

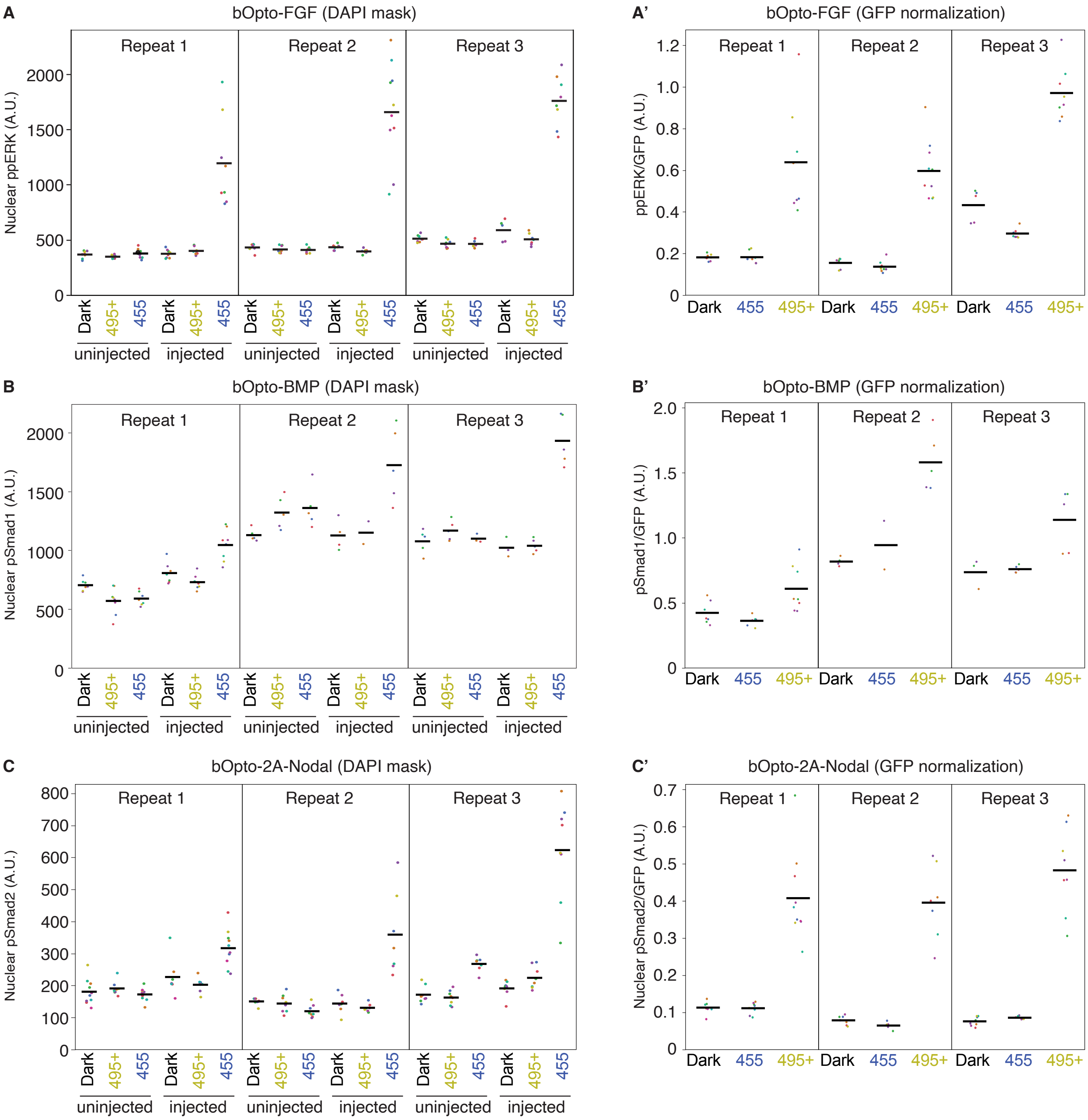

Supplementary Figure 3: Wavelength-dependent activation of FGF, BMP, & Nodal signaling quantification I. A-C’) Embryos injected at the one-cell stage with the indicated mRNA (+ *GFP* mRNA) were exposed to dark, 495+ nm light (18.51 W/m^2), or 455 nm light (50 W/m^2) starting at early gastrulation (50% epiboly - shield) for 30 min. HCR-IF was used to detect activated signaling effectors (ppERK, pSmad1, and pSmad2 reflect FGF, BMP, and Nodal signaling, respectively). A,B,C) Raw phosphorylated signaling effector intensity was measured in each DAPI-positive nuclear pixel. Each dot represents the median nuclear pixel intensity in one embryo. Black lines represent the mean nuclear intensity of all embryos in the indicated condition. A’, B’) GFP-normalized phosphorylated effector signal in each GFP-positive pixel. Each dot represents the median GFP-normalized pixel signal in one embryo. Black lines represent the mean GFP-normalized signal of all embryos in the indicated condition. C’) pSmad2 intensity in each DAPI + GFP-positive pixel was divided by the corresponding GFP intensity. Each dot represents the median GFP-normalized nuclear pixel signal in one embryo. Black lines represent the mean GFP-normalized nuclear signal of all embryos in the indicated condition.

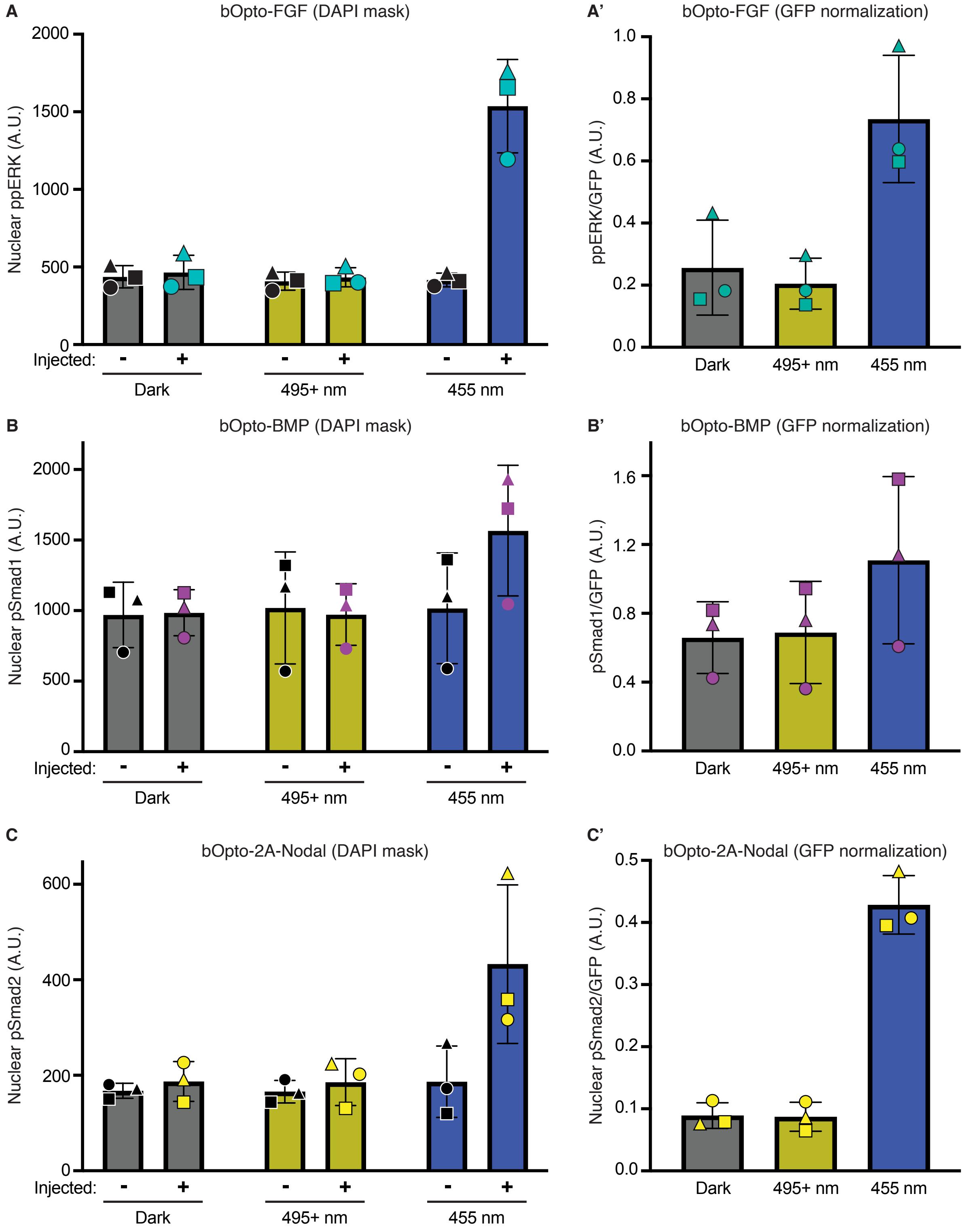

Supplementary Figure 4: Wavelength-dependent activation of FGF, BMP, & Nodal signaling quantification II. Uninjected embryos (-) and embryos injected (+) at the one-cell stage with the indicated mRNA + *GFP* mRNA were exposed to dark, 495+ nm light, or 455 nm light starting at early gastrulation (50% epiboly - shield) for 30 min. HCR-IF was used to detect phosphorylated signaling effectors (ppERK, pSmad1, and pSmad2 reflect FGF, BMP, and Nodal signaling, respectively). **A,B,C)** Raw phosphorylated signaling effector intensity was measured in each DAPI-positive nuclear pixel. Each symbol (square, circle, triangle) represents the averaged median intensities from all embryos in one experimental repeat. **A',B')** Phosphorylated signaling effector intensity in each GFP-positive pixel was divided by the corresponding GFP intensity. Each symbol (square, circle, triangle) represents the averaged median GFP-normalized signal from all embryos in one experimental repeat. **C')** pSmad2 intensity in each DAPI + GFP-positive pixel was divided by the corresponding GFP intensity. Each symbol (square, circle, triangle) represents the averaged median GFP-normalized signal from all embryos in one experimental repeat.

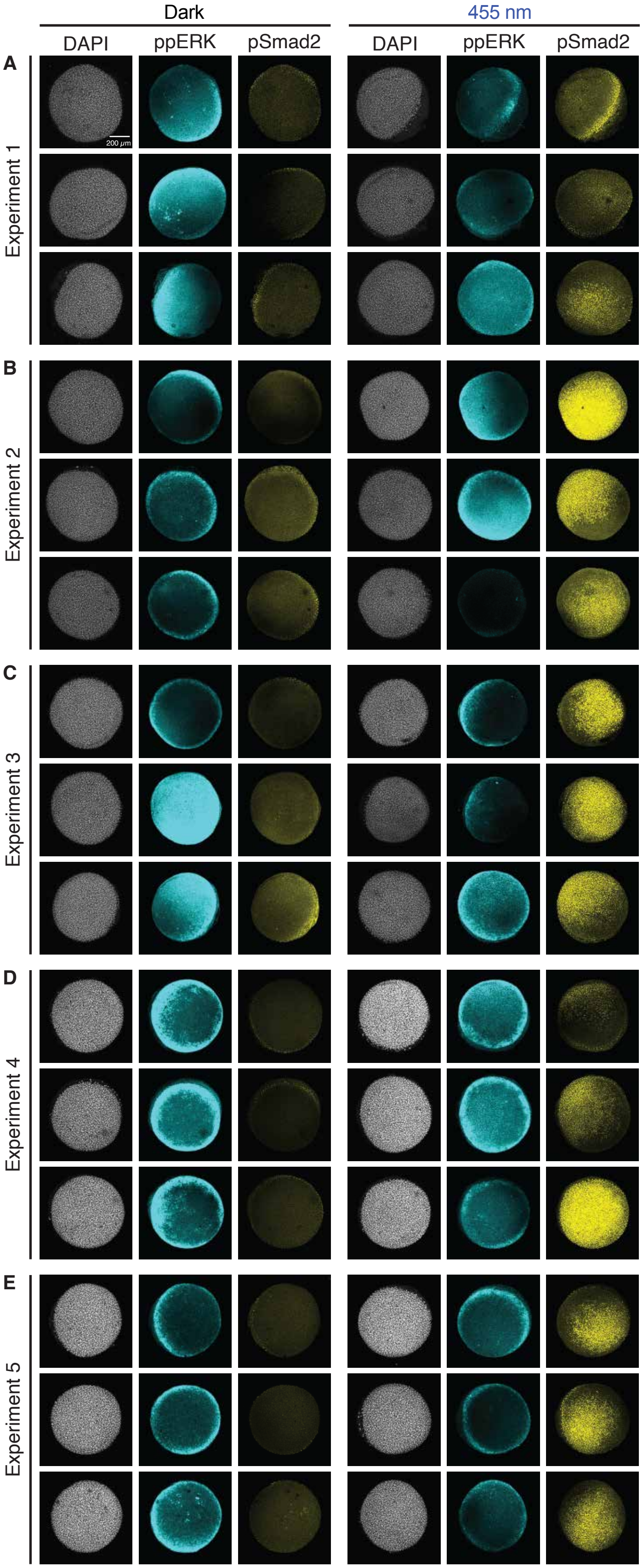

Supplementary Figure 5: bOpto-2A-Nodal triple immunofluorescence repeats. A-E) Embryos injected at the one-cell stage with mRNA encoding *bOpto-2A-Nodal* exposed to dark (left panel) or 455 nm light (50 W/m^2, right panel) starting at early gastrulation (50% epiboly - shield stage) for 30 min. Triple IF staining was used to simultaneously detect activated signaling effectors (pSmad1, ppERK, and pSmad2 reflecting BMP, FGF, and Nodal signaling, respectively). Only ppERK and pSmad2 shown here. Three bOpto-2A-Nodal-expressing embryos per condition from five experimental repeats are shown. (Scale bar is 200 μm).

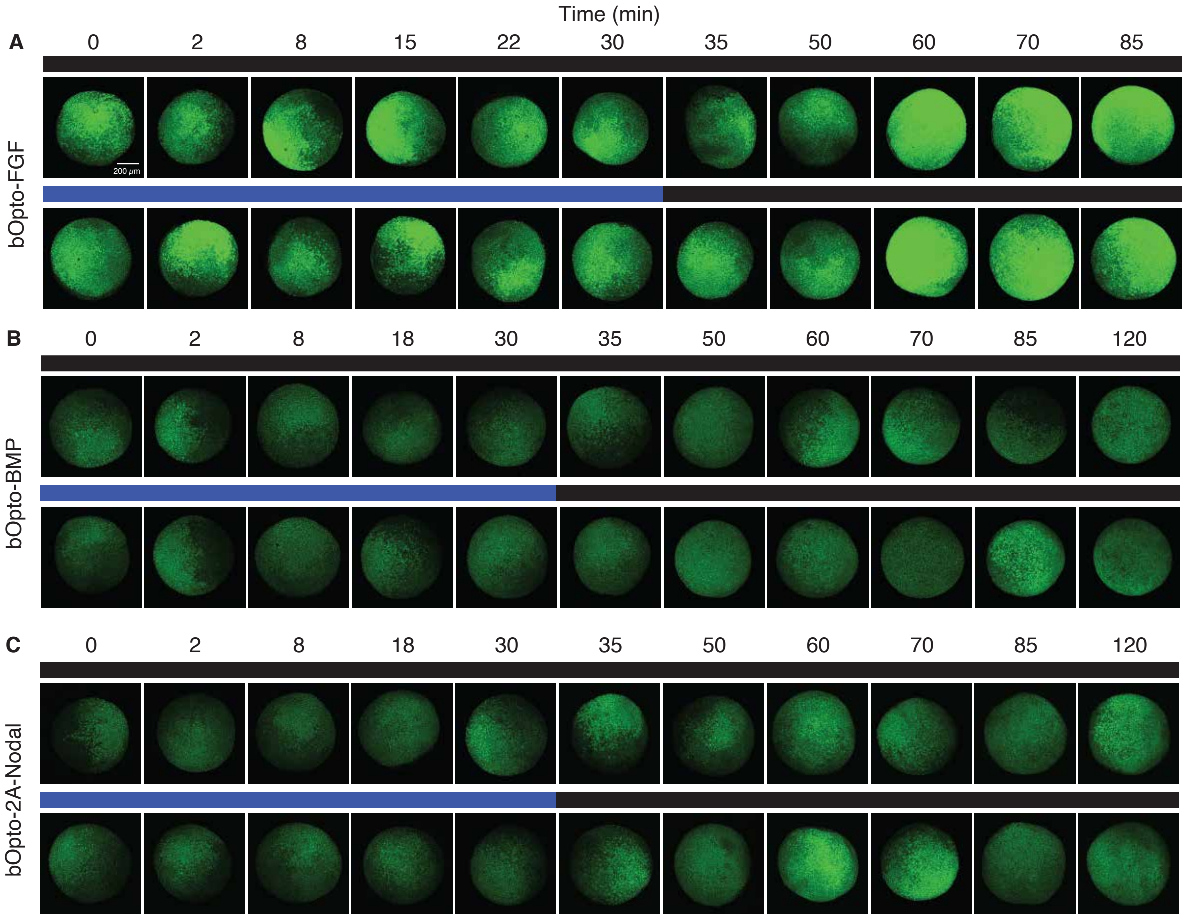

Supplementary Figure 6: Co-injected GFP signal used for quantification of on / off kinetics. A-C) Embryos were injected at the one-cell stage with mRNA encoding *GFP* and either *bOpto-FGF* (A), *bOpto-BMP* (B), or *bOpto-2A-Nodal* (C). Starting at early gastrulation (50% epiboly - shield), embryos were exposed to 455 nm light (50 W/m^2) for 30 min and fixed at different time points during and after exposure. HCR-IF staining used to detect activated signaling effectors is shown in Fig. 5 (ppERK, pSmad1, and pSmad2 reflect FGF, BMP, and Nodal signaling, respectively). HCR-IF signal was normalized voxel-wise based on co-injected GFP intensity shown here (see Supp. Fig. 7). GFP images correspond to representative IF images shown in Fig. 5. (Scale bar is 200 μm).

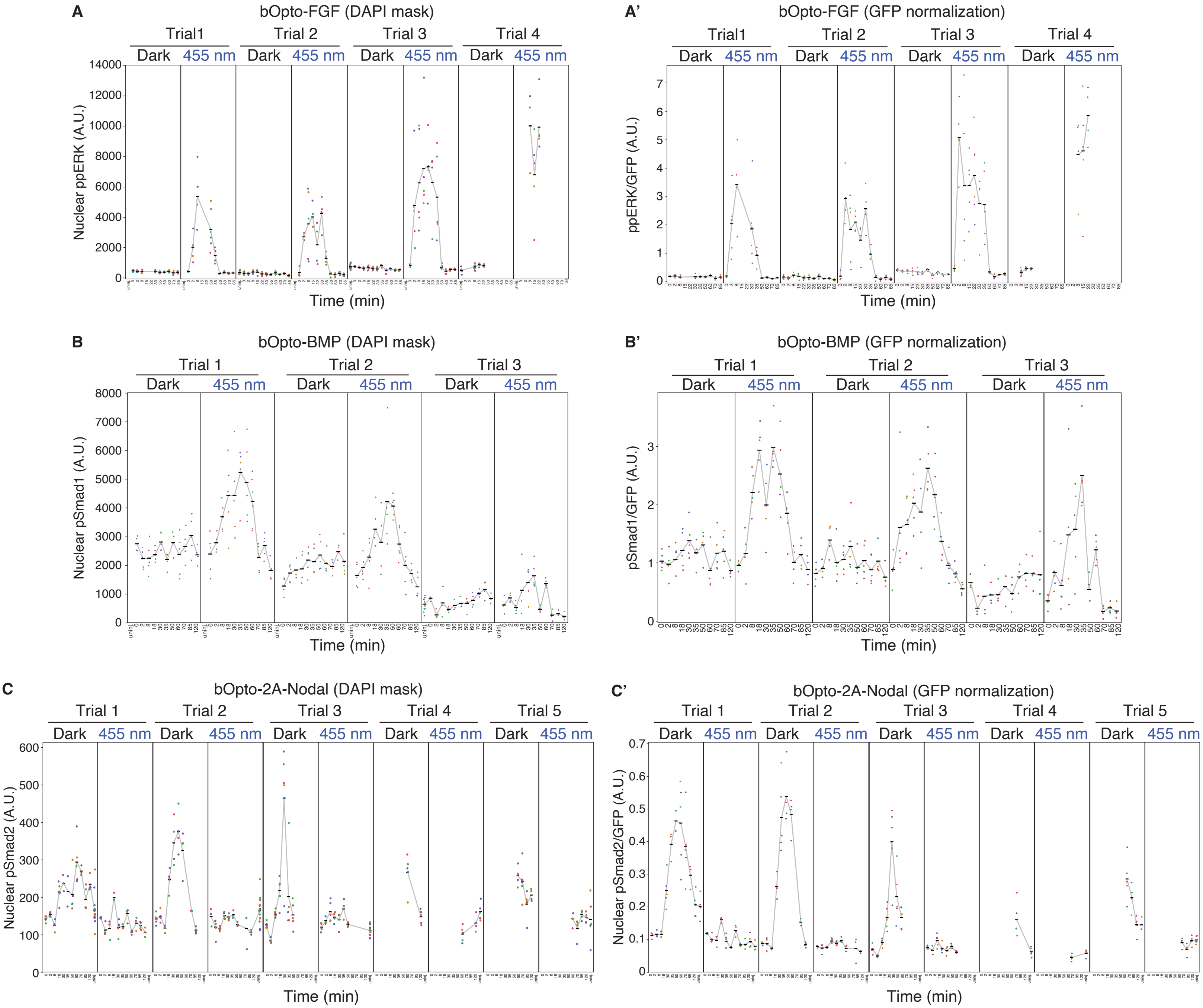

Supplementary Figure 7: Quantification of optogenetic signaling activator toolkit on/off kinetics. Embryos were injected at the one-cell stage with mRNA encoding *GFP* and either *bOpto-FGF* (A,A’), *bOpto-BMP* (B,B’), or *bOpto-2A-Nodal* (C,C’). Starting at early gastrulation (50% epiboly - shield), embryos were exposed to 455 nm light (50 W/m^2) for 30 min and fixed during and after exposure. Three full repeats were performed across 3-5 trials. HCR-IF staining used to detect phosphorylated signaling effectors is shown in Fig. 5 (ppERK, pSmad1, and pSmad2 reflect FGF, BMP, and Nodal signaling, respectively). **A,B,C)** Raw phosphorylated signaling effector intensity was measured in each DAPI-positive nuclear pixel. Each dot represents the median nuclear pixel intensity in one embryo. Black lines represent the mean nuclear intensity of all embryos in the indicated condition. **A’,B’)** Phosphorylated siganling effector intensity in each GFP-positive pixel was divided by the corresponding GFP intensity. Each dot represents the median GFP-normalized pixel signal in one embryo. Black lines represent the mean GFP-normalized signal of all embryos in the indicated condition. **C’)** pSmad2 intensity in each DAPI + GFP-positive pixel was divided by the corresponding GFP intensity. Each dot represents the median GFP-normalized nuclear pixel signal in one embryo. Black lines represent the mean GFP-normalized nuclear signal of all embryos in the indicated condition.

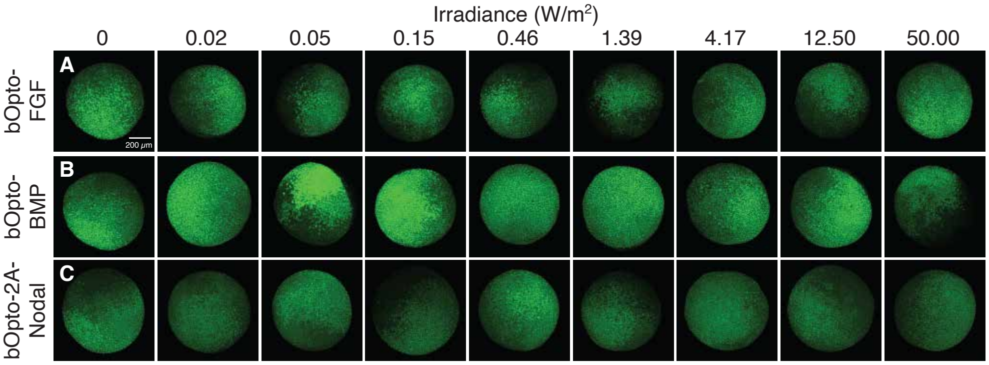

Supplementary Figure 8: Co-injected GFP signal used for quantification of irradiance dependence HCR-IF. A-C) Embryos were injected at the one-cell stage with mRNA encoding *GFP* and either *bOpto-FGF* (A), *bOpto-BMP* (B), or *bOpto-2A-Nodal* (C). Starting at early gastrulation (50% epiboly - shield), embryos were exposed to 455 nm light (50 W/m^2) at the indicated irradiances for 5 min (bOpto-FGF) or 25 min (bOpto-BMP and -2A-Nodal). HCR-IF staining used to detect phosphorylated signaling effectors is shown in Fig. 6 (ppERK, pSmad1, and pSmad2 to reflect FGF, BMP, and Nodal signaling, respectively). HCR-IF signal was normalized based on co-injected GFP intensity shown here; GFP images correspond to representative images shown in Fig. 6. (Scale bar is 200 μm).

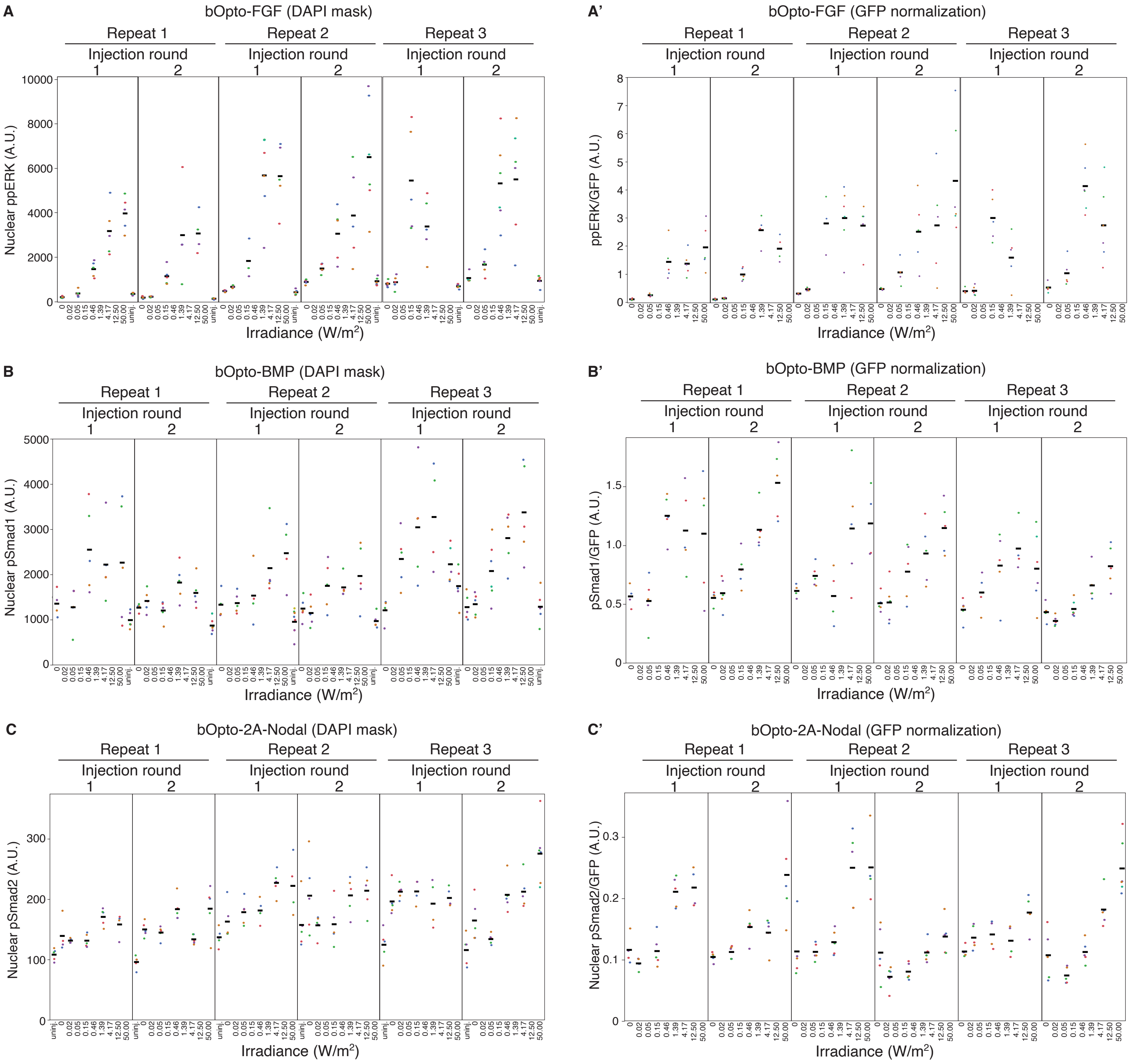

Supplementary Figure 9: Quantification of light irradiance dependence. Embryos were injected at the one-cell stage with mRNA encoding *GFP* and either *bOpto-FGF* (A,A'), *bOpto-BMP* (B,B'), or *bOpto-2A-Nodal* (C,C'). At early gastrulation (50% epiboly - shield), embryos were exposed to 455 nm light (50 W/m^2) at the indicated irradiance for 5 min (bOpto-FGF) or 25 min (bOpto-BMP and -2A-Nodal). HCR-IF was used to detect phosphorylated signaling effectors (ppERK, pSmad1, and pSmad2 reflect FGF, BMP, and Nodal signaling, respectively). **A,B,C)** Raw phosphorylated signaling effector intensity was measured in each DAPI-positive nuclear pixel. Each dot represents the median nuclear pixel intensity in one embryo. Black lines represent the mean nuclear intensity of all embryos in the indicated condition. **A',B')** Phosphorylated siganling effector intensity in each GFP-positive pixel was divided by the corresponding GFP intensity. Each dot represents the median GFP-normalized pixel signal in one embryo. Black lines represent the mean GFP-normalized signal of all embryos in the indicated condition. **C')** pSmad2 intensity in each DAPI + GFP-positive pixel was divided by the corresponding GFP intensity. Each dot represents the median GFP-normalized nuclear pixel signal in one embryo. Black lines represent the mean GFP-normalized nuclear signal of all embryos in the indicated condition.

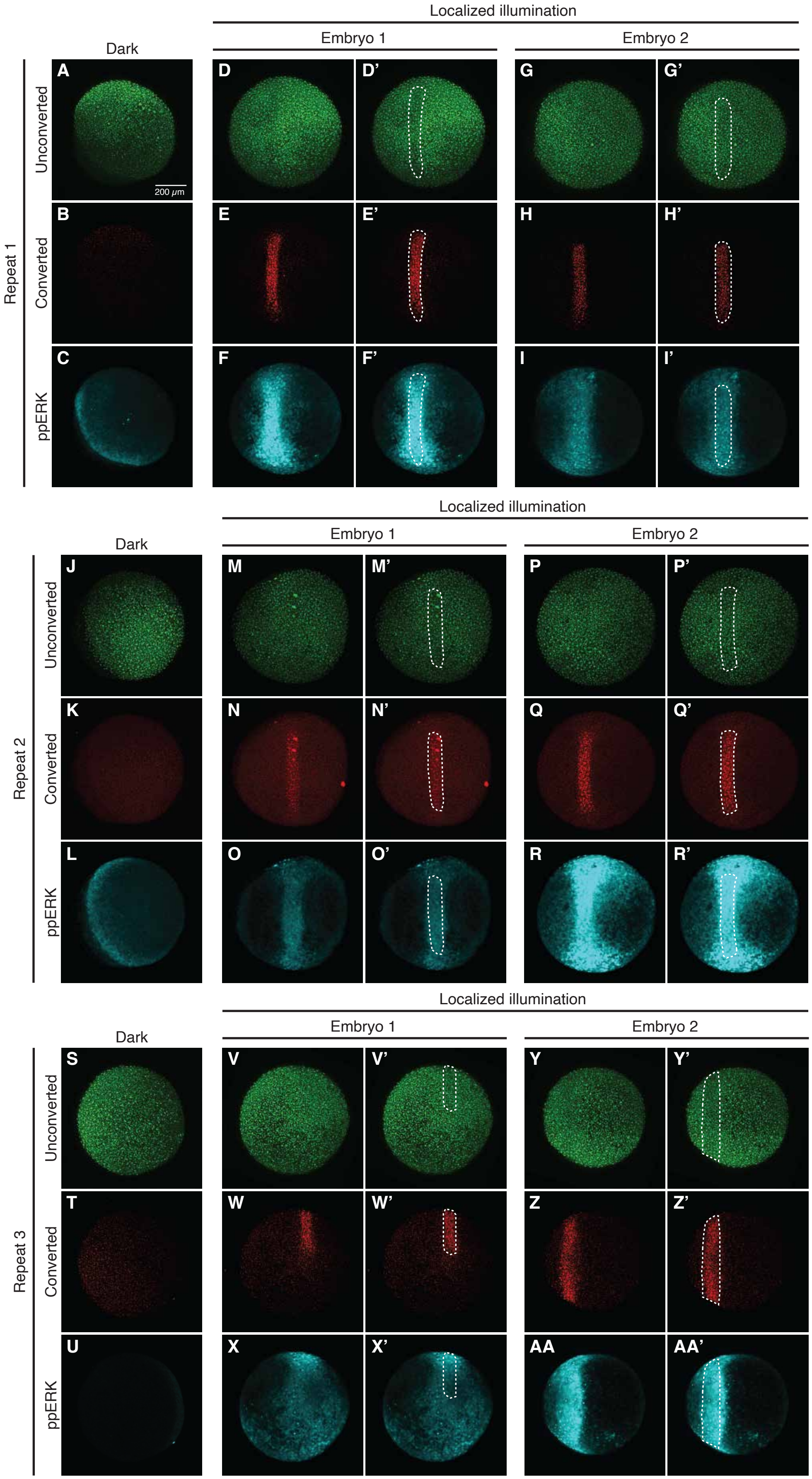

Supplementary Figure 10: Spatially localized FGF signaling activation repeats. Embryos were injected at the one-cell stage with mRNA encoding the green-to-red photoconvertible fluorescent proteins *nls-Kaede* and *bOpto-FGF*. At early gastrulation embryos were either kept in the dark (**A-C, J-L, S-U**) or illuminated locally with 405 and 445 nm confocal lasers. HCR-IF was used to detect ppERK. Two representative exposed embryos from three repeats shown (**D-I', M-R', V-AA'**, respectively). Dotted white lines outline photoconverted Kaede from images to the left. A-F' are shown in Fig. 7. (Scale bar is 200 μm).

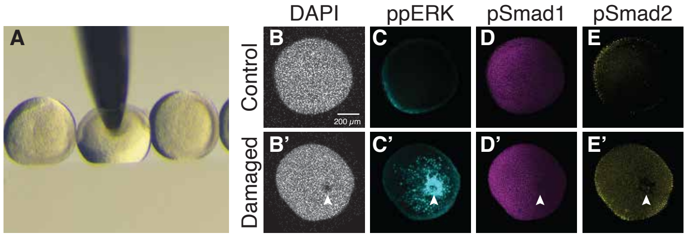

Supplementary Figure 11: Damage-activated ERK phosphorylation at gastrulation stage. A-E) Wild type, uninjected zebrafish embryos were dechorionated with pronase at the one-cell stage. At early gastrulation (~50% epiboly), embryos were left undamaged (B,C,D,E) or firmly poked with a metal probe (A,B',C',D',E') and fixed 20 min later. Triple IF + DAPI staining was used to detect nuclei (B, B') and phosphorylated signaling effectors ppERK (C,C'), pSmad1 (D,D') and, pSmad2 (E,E'). Damage visible in the DAPI channel correlates with ERK phosphorylation (arrowheads). (Scale bar is 200 μm).

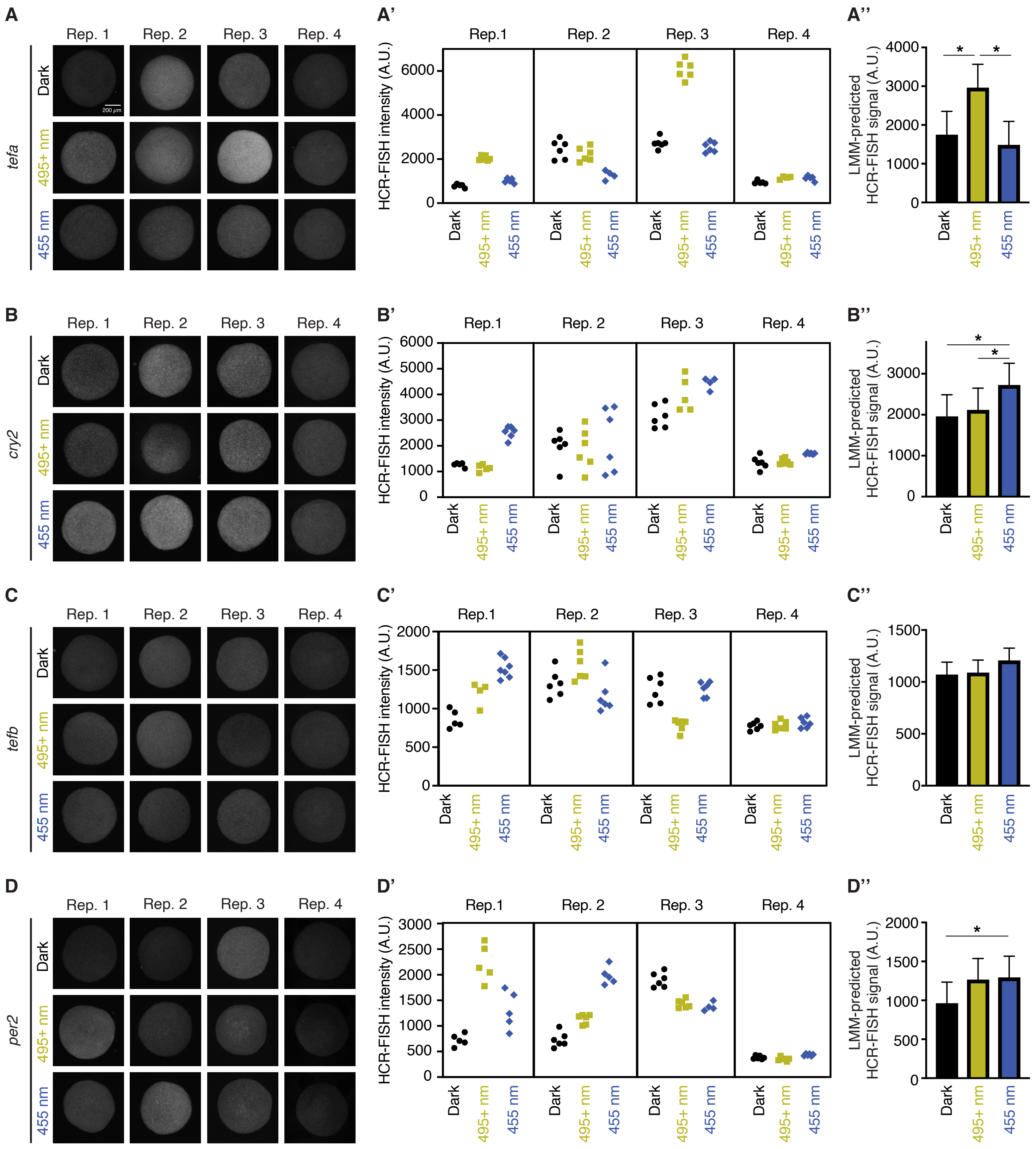

Supplementary Figure 12: Circadian gene responses to light exposure in gasturation stage embryos. A-D) Uninjected AB wild type zebrafish embryos were reared in the dark. At early gastrulation (shield stage), a subset of embryos were exposed to 495+ nm light (18.51 W/m^2) or 455 nm light (50 W/m^2) for 30 min, then dark for 20 min. A third set of embryos were maintained in the dark throughout the experiment. HCR-FISH was performed for the indicated circadian-related genes. Each panel represents four experimental repeats for the indicated gene. (Scale bar is 200 μm). **A'-D')** Mean HCR-FISH intensities from experiments shown in A-D. Each symbol represents mean intensity from one embryo. **A''-D'')** Quantification of experiments shown in A-D. Linear-mixed model-predicted least squared means of HCR-FISH signal +/- SEM (N = 4; LMM - Fixed Effect: Wavelength, Random Effect: Replicate; post hoc Tukey's HSD where * indicates $p < 0.05$).
