# Supplementary material for: An optogenetic toolkit for robust activation of FGF, BMP, & Nodal signaling in zebrafish
Source: bioRxiv. 2025 Apr 19:2025.04.17.649426. Preprint. [Version 1] doi: 10.1101/2025.04.17.649426 (PMC12190770; doi:10.1101/2025.04.17.649426)
Supplement: Supplement 1 [file media-1.zip › Supplementary_Materials/Supplementary_Materials.pdf]

SUPPLEMENTARY MATERIALS

Supplementary Schematics

Supplementary Schematic 1: On/off kinetics of optogenetic signaling activator toolkit I.

Supplementary Tables

Supplementary Table 1: Summary of Imaging Parameters.

Supplementary Table 2: Wavelength-dependent activation of FGF, BMP, and Nodal signaling I.

Supplementary Table 3: Wavelength-dependent activation of FGF, BMP, and Nodal signaling II.

Supplementary Table 4: Wavelength-dependent activation of FGF, BMP, and Nodal signaling III.

Supplementary Table 5: Pathway specific optogenetic activation of FGF, BMP, and Nodal signaling I.

Supplementary Table 6: On/off kinetics of optogenetic signaling activator toolkit I.

Supplementary Table 7: On/off kinetics of optogenetic signaling activator toolkit II.

Supplementary Table 8: On/off kinetics of optogenetic signaling activator toolkit III.

Supplementary Table 9: On/off kinetics of optogenetic signaling activator toolkit IV.

Supplementary Table 10: Irradiance sensitivity of optogenetic toolkit I.

Supplementary Table 11: List of pathway-specific target genes and corresponding HCR initiators and amplifiers.

Supplementary Table 12: List of pathway-specific target genes and corresponding HCR probes.

Supplementary Information

Supplementary Information 1: Construct sequences.

References

Supplementary Schematic 1: On/off kinetics of optogenetic signaling activator toolkit I. Visualization of tON & tOFF parameters to describe on/off kinetics in Fig. 5.

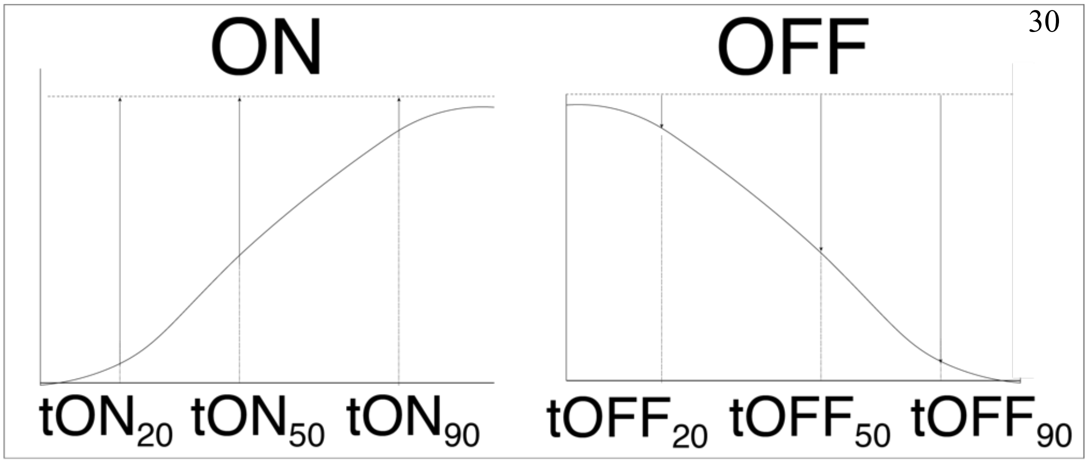

Supplementary Table 1: Summary of Imaging Parameters. Fluorophores used in HCR-IF, HCR-FISH, Triple-IF and fluorescent proteins detected with corresponding imaging conditions.

**Fluorophore/Protein**	**Fluorophore/Fluorescent protein**	**Laser Line (nm)**	**Emission wavelength (nm)**	**Signal**	**Experiments**
Alexa-647	Fluorophore	640	553-700	pSmad1, pSmad2, ppERK1/2, s*izzled, bambia, spry4, il17rd, noto, lft1*	Time Course, Intensity Dependent, Wavelength, Gene Expression, Spatial Activation
Alexa-546	Fluorophore	561	553-700	pSmad2, *gata2a, dusp6, gsc*	Intensity Dependent, Gene Expression
Kaede-Red	Fluorescent protein	561	576-616	Nuclear-Kaede-Red	Spatial Activation
Cy3	Fluorophore	561	560-700	ppERK1/2	Pathway Dependence
FITC	Fluorophore	488	480-639	pSmad2	Pathway Dependence
Alexa-488	Fluorophore	488	480-560	*sizzled, bambia, spry4, il17rd, noto, lft1*	Gene Expression
GFP	Fluorescent protein	488	480-560	GFP	Time Course, Intensity Dependence, Wavelength
Kaede-Green	Fluorescent protein	488	410-546	Nuclear-Kaede-Green	Spatial Activation
DAPI	Fluorophore	405	400-489	Nuclei	Used in all experiments except Spatial experiment

Supplementary Table 2: Wavelength-dependent activation of FGF, BMP, and Nodal signaling I. Fixed effects tests from Linear Mixed-effects Model (LMM) shown in Fig. 2. Fixed Effect: Wavelength, Random Effect: Biological Replicate; Personality: Standard Least Squares, Method: REML. $p < 0.05$ considered to be significant.

	Source	Nparm	DF	DFDen	F Ratio	Prob > F
**FGF**	Exposure	2	2	65.01	143.85	<.0001
**BMP**	Exposure	2	2	41.03	26.58	<.0001
**Nodal**	Exposure	2	2	65.08	188.71	<.0001

Supplementary Table 3: Wavelength-dependent activation of FGF, BMP, and Nodal signaling II. Least square means estimates from Linear Mixed-effects Model (LMM) shown in Fig. 2 and Supp. Table 2. Fixed Effect: Wavelength, Random Effect: Biological Replicate; Personality: Standard Least Squares, Method: REML.

	Exposure	Estimate	Std Error	DF	Lower 95%	Upper 95%
**FGF**	Dark	0.25	0.09	2.26	-0.09	0.60
	Yellow	0.21	0.09	2.22	-0.14	0.55
	Blue	0.73	0.09	2.18	0.38	1.08
**BMP**	Dark	0.68	0.20	2.17	-0.13	1.50
	Yellow	0.71	0.20	2.18	-0.10	1.51
	Blue	1.09	0.20	2.11	0.26	1.91
**Nodal**	Dark	0.09	0.02	10.26	0.05	0.12
	Yellow	0.09	0.02	9.57	0.05	0.12
	Blue	0.43	0.02	8.31	0.39	0.46

Supplementary Table 4: Wavelength-dependent activation of FGF, BMP, and Nodal signaling III. Results of *post hoc* pairwise comparisons of Least square means and standard errors estimated in Supp. Table 2-3. Comparisons were performed with Tukey's HSD test where $p < 0.05$ is considered to be significant and denoted with * in Fig. 2.

<table>
<tr><th></th><th>Exposure</th><th>Minus Exposure</th><th>Difference</th><th>Std Error</th><th>t Ratio</th><th>Prob>|t|</th><th>Lower 95%</th><th>Upper 95%</th></tr>
<tr><td>**FGF**</td><td>Dark</td><td>Yellow</td><td>0.05</td><td>0.04</td><td>1.28</td><td>0.41</td><td>-0.04</td><td>0.13</td></tr>
<tr><td></td><td>Dark</td><td>Blue</td><td>-0.48</td><td>0.04</td><td>-13.55</td><td><.0001</td><td>-0.56</td><td>-0.39</td></tr>
<tr><td></td><td>Yellow</td><td>Blue</td><td>-0.52</td><td>0.03</td><td>-15.27</td><td><.0001</td><td>-0.61</td><td>-0.44</td></tr>
<tr><td>**BMP**</td><td>Dark</td><td>Yellow</td><td>-0.02</td><td>0.07</td><td>-0.31</td><td>0.95</td><td>-0.19</td><td>0.14</td></tr>
<tr><td></td><td>Dark</td><td>Blue</td><td>-0.40</td><td>0.06</td><td>-6.35</td><td><.0001</td><td>-0.56</td><td>-0.25</td></tr>
<tr><td></td><td>Yellow</td><td>Blue</td><td>-0.38</td><td>0.06</td><td>-5.97</td><td><.0001</td><td>-0.54</td><td>-0.23</td></tr>
<tr><td>**Nodal**</td><td>Dark</td><td>Yellow</td><td>0.00</td><td>0.02</td><td>0.02</td><td>1.00</td><td>-0.05</td><td>0.05</td></tr>
<tr><td></td><td>Dark</td><td>Blue</td><td>-0.34</td><td>0.02</td><td>-16.56</td><td><.0001</td><td>-0.39</td><td>-0.29</td></tr>
<tr><td></td><td>Yellow</td><td>Blue</td><td>-0.34</td><td>0.02</td><td>-16.82</td><td><.0001</td><td>-0.39</td><td>-0.29</td></tr>
</table>

Supplementary Table 5: Pathway specific optogenetic activation of FGF, BMP, and Nodal signaling I. Minimum and maximum values used in the Fiji-macro (see Codes folder, "Pathway_Specific_Activation_Macro.ijm") to generate maximum intensity projections (MIPs) seen in Fig. 4.

Pathway	Signal	Minimum	Maximum
**BMP**	DAPI	85	47000
	pSmad2	1000	9300
	ppERK	80	7500
	pSmad1	80	2500
**FGF**	DAPI	85	47000
	pSmad2	1000	9300
	ppERK	80	3500
	pSmad1	80	2500
**Nodal**	DAPI	85	4700
	pSmad2	1000	10000
	ppERK	80	40000
	pSmad1	80	6000

Supplementary Table 6: On/off kinetics of optogenetic signaling activator toolkit I. Fixed effects tests from One-way ANOVA shown in Fig. 5. Fixed Effect: Time. $p < 0.05$ was considered to be significant.

Pathway	Source	DF	Sum of Squares	Mean Square	F Ratio	Prob > F
**FGF**	Time	10	70.10	7.01	7.94	<.0001
	Error	23	20.30	0.88		
	C. Total	33	90.40			
**BMP**	Time	10	13.13	1.31	8.95	<.0001
	Error	22	3.22	0.15		
	C. Total	32	16.35			
**Nodal**	Time	10	0.51	0.05	7.84	<.0001
	Error	22	0.14	0.01		
	C. Total	32	0.65			

Supplementary Table 7: On/off kinetics of optogenetic signaling activator toolkit II. LSD Threshold Matrix of pairwise *post hoc* comparisons to time = 0 min (Fig. 5 and Supp. Table 6). Comparisons were performed with Dunnett's Method for multiple comparison where $p < 0.05$ is considered to be significant and denoted with * in Fig. 5.

FGF			rimBMP			Nodal		
**Time**	**Abs(Dif)-LSD**	**p-Value**	**Time**	**Abs(Dif)-LSD**	**p-Value**	**Time**	**Abs(Dif)-LSD**	**p-Value**
0	-2.26	1	0	-0.92	1	0	-0.19	1
2	0.83	0.0042	2	-0.31	0.3259	2	-0.19	1
8	0.87	0.0031	8	-0.28	0.2705	8	-0.19	1
15	0.74	0.0056	18	0.45	0.0019	18	-0.08	0.4192
22	1.09	0.0019	30	0.04	0.039	30	0.15	0.0003
30	-0.10	0.0666	35	0.87	<.0001	35	0.13	0.0005
35	-0.99	0.5004	50	0.03	0.0409	50	0.09	0.0023
50	-2.24	1	60	-0.22	0.1993	60	0.01	0.0358
60	-2.15	1	70	-0.79	0.9998	70	-0.04	0.1815
70	-2.23	1	85	-0.75	0.9978	85	-0.13	0.94
85	-2.23	1	120	-0.77	0.9991	120	-0.14	0.9539

Supplementary Table 8: On/off kinetics of optogenetic signaling activator toolkit III.

Parameters predicted with 95% CI from non-linear, three-parameter logistic regression of data in Fig. 5A''-C''; Data from Fig.5A'-C' & Supp. Fig. where time <= 30 min.

Pathway	Phase		R2	Growth Rate	Inflection Point	Asymptote	tON20	tON50	tON90
**FGF**	**ON**	Predictive Y					0.59	1.47	2.65
		Estimate	0.503	10.85	0.42	2.95	0.29	0.42	0.62
		Lower 95% CI		-6951892.81	-267998.42	2.32	-186110.00	-267998.00	-397788.00
		Upper 95% CI		6951914.51	267999.25	3.57	186111.00	267999.20	397789.00
**BMP**	**ON**	Predictive Y					0.21	0.52	0.94
		Estimate	0.534	0.30	6.36	1.05	1.78	6.36	13.62
		Lower 95% CI		-0.14	0.47	0.68	-4.97	1.35	0.75
		Upper 95% CI		0.74	12.26	1.41	8.53	11.37	26.49
**Nodal**	**ON**	Predictive Y					0.07	0.17	0.31
		Estimate	0.966	0.38	19.78	0.35	16.12	19.78	25.59
		Lower 95% CI		-0.41	15.08	0.27	12.25	16.13	14.04
		Upper 95% CI		1.16	24.48	0.42	19.99	23.43	37.14

Supplementary Table 9: On/off kinetics of optogenetic signaling activator toolkit IV.

Parameters predicted with 95% CI from non-linear, three-parameter logistic regression of data in Fig. 5A’’’-C’’’; Data from Fig.5A’-C’ where time >= 30 min.

Pathway	Phase		R2	Growth Rate	Inflection Point	Asymptote	tOFF20	tOFF50	tOFF90
**FGF**	**OFF**	Predictive Y					1.97	1.23	0.25
		Estimate	0.877	-0.39	35.30	2.46	1.76	5.30	10.92
		Lower 95% CI		-4.04	23.27	-4.15	-9.05	2.72	-37.37
		Upper 95% CI		3.26	47.33	9.07	12.56	7.88	59.20
**BMP**	**OFF**	Predictive Y					0.98	0.61	0.12
		Estimate	0.667	-0.22	57.11	1.22	20.81	27.11	37.10
		Lower 95% CI		-0.57	48.41	0.82	8.47	19.56	20.99
		Upper 95% CI		0.13	65.82	1.63	33.15	34.67	53.21
**Nodal**	**OFF**	Predictive Y					0.31	0.19	0.04
		Estimate	0.626	-0.06	62.67	0.39	10.63	32.67	67.60
		Lower 95% CI		-0.14	36.80	0.15	-4.34	22.22	33.56
		Upper 95% CI		0.01	88.54	0.62	25.61	43.12	101.63

Supplementary Table 10: Irradiance sensitivity of optogenetic toolkit I. Parameters predicted with 95% CI from non-linear, three-parameter logistic regression of data in Fig. 6B'-D'. Dosage parameters (D20 & D90) are calculated from corresponding Irradiance parameters (I20, I90) by multiplying by time of exposure in seconds for each tool (FGF: 5 min, BMP/Nodal: 25 min). Fold increase is a ratio between the D20 & D90 of each tool & FGF's D20 and D90, respectively.

Pathway		R2	Growth Rate	Inflection Point	Asymptote				Dosage		Fold Increase over FGF	
						I20	I50	I90	D20	D90	D20	D90
**FGF**	Predictive Y					0.45	1.11	2.00				
	Estimate	0.748	37.40	0.09	2.23	0.05	0.09	0.15	16.44	27.56	1.00	1.00
	Lower 95% CI		-2.68	0.03	1.88	0.00	0.03	0.05	0.54	9.52		
	Upper 95% CI		77.47	0.15	2.57	0.11	0.15	0.25	32.34	45.59		
**BMP**	Predictive Y					0.10	0.26	0.47				
	Estimate	0.678	6.96	0.35	0.52	0.16	0.35	0.67	232.69	1005.54	14.00	36.00
	Lower 95% CI		1.00	0.16	0.43	-0.03	0.17	0.26	-52.48	396.34		
	Upper 95% CI		12.91	0.55	0.62	0.35	0.53	1.08	517.87	1614.73		
**Nodal**	Predictive Y					0.03	0.07	0.12				
	Estimate	0.578	0.20	11.00	0.13	3.90	11.00	22.25	5845.05	33376.65	356.00	1211.00
	Lower 95% CI		0.05	4.57	0.09	-0.27	5.54	6.12	-406.04	9182.01		
	Upper 95% CI		0.34	17.42	0.18	8.06	16.45	38.38	12096.15	57571.22		

Supplementary Table 11: List of pathway-specific target genes and corresponding HCR initiators and amplifiers. Used to generate images in Fig. 3.

Pathway	Gene	Initiator	Amplifier
BMP	*bambia*	B1	647
	gata2a	B2	546
	szl	B3	488
FGF	*spry4*	B1	647
	dusp6	B2	546
	Il17rd	B3	488
Nodal	*noto*	B1	647
	gsc	B2	546
	lft1	B3	488

Supplementary Table 12: List of pathway-specific target genes and corresponding HCR probes. Used to generate images in Fig. 3.

Sequence name	Sequence
B1_bambia_26_Dla50	GAGGAGGGCAGCAAACGGaaCATAAGCTCCACCCTTTTCATATTC
B1_bambia_26_Dla50	AGAGGGAAGGAGGTGGGGCTTGCAGtaGAAGAGTCTTCCTTTACG
B1_bambia_26_Dla50	GAGGAGGGCAGCAAACGGaaCCTTGAAATGTCTTTGCTCTCGTTG
B1_bambia_26_Dla50	TTTTTGAGAAAACACAATGAATTGTtaGAAGAGTCTTCCTTTACG
B1_bambia_26_Dla50	GAGGAGGGCAGCAAACGGaaAACATTACGACAGCAAACTACACAG
B1_bambia_26_Dla50	ACAATAAAATAACACGCCGATAATAtaGAAGAGTCTTCCTTTACG
B1_bambia_26_Dla50	GAGGAGGGCAGCAAACGGaaTGAGTAGTCATACGAACTCCAGCTT
B1_bambia_26_Dla50	CCCTTTTGTGCCCTAACAAAGAAGCtaGAAGAGTCTTCCTTTACG
B1_bambia_26_Dla50	GAGGAGGGCAGCAAACGGaaCAGAGATAGGAGACGCTCACCCCCG
B1_bambia_26_Dla50	GTGCCCCGTGTACATCCCCCAGTGTtaGAAGAGTCTTCCTTTACG
B1_bambia_26_Dla50	GAGGAGGGCAGCAAACGGaaCGCAGCTTATCGCAGCCCAGACAAC
B1_bambia_26_Dla50	CCTCCTCCAGTGCACAAATCCGTCTtaGAAGAGTCTTCCTTTACG
B1_bambia_26_Dla50	GAGGAGGGCAGCAAACGGaaACTCCAAGTCCAACTTAGCCACGTG
B1_bambia_26_Dla50	TCTCATGTCCCGTTACCGGCACCATtaGAAGAGTCTTCCTTTACG
B1_bambia_26_Dla50	GAGGAGGGCAGCAAACGGaaGCTGTAATGCAGGCGAGAAAGCATC
B1_bambia_26_Dla50	TTTCTTGGCATGGTGGTGTCCGTGAtaGAAGAGTCTTCCTTTACG
B1_bambia_26_Dla50	GAGGAGGGCAGCAAACGGaaTCGCTACGGAGCATTCGCAACGCCA
B1_bambia_26_Dla50	TGGCGCTGTGCCTGGAGACGCTTGTtaGAAGAGTCTTCCTTTACG
B1_bambia_26_Dla50	GAGGAGGGCAGCAAACGGaaCCGCGATGGGAACCGCTATCACCGC
B1_bambia_26_Dla50	TAATCAGCAGAACCAGGATAAGCCCtaGAAGAGTCTTCCTTTACG
B1_bambia_26_Dla50	GAGGAGGGCAGCAAACGGaaTAACTCTTGCACCCTTGTGATCAGG
B1_bambia_26_Dla50	CCGGAACCACACCTCTTTAGCAGACtaGAAGAGTCTTCCTTTACG
B1_bambia_26_Dla50	GAGGAGGGCAGCAAACGGaaGAGTCCCCTCTGGGGTGTGTGAGGT
B1_bambia_26_Dla50	TGATTGGAGCTGTGGTATCGGTCTGtaGAAGAGTCTTCCTTTACG
B1_bambia_26_Dla50	GAGGAGGGCAGCAAACGGaaCATGGCAGCACTCCACAGGAGAGGA
B1_bambia_26_Dla50	GCAAACCCCTGTAGTTACACATATCtaGAAGAGTCTTCCTTTACG
B1_bambia_26_Dla50	GAGGAGGGCAGCAAACGGaaAGAGCACACGTCTGCAGAGTTTAAA
B1_bambia_26_Dla50	TCCACTTGAAATGTCCACATTTTTAtaGAAGAGTCTTCCTTTACG
B1_bambia_26_Dla50	GAGGAGGGCAGCAAACGGaaGAGTTTGTGTTAAGAGGGTCCAGGA
B1_bambia_26_Dla50	GAATCCACGCAGCCGTGTGTTAAAGtaGAAGAGTCTTCCTTTACG
B1_bambia_26_Dla50	GAGGAGGGCAGCAAACGGaaTACACATGTATCCGGTGGCAACGCA
B1_bambia_26_Dla50	TAGTAAAGCAAGCGTTGAGCTCTGAtaGAAGAGTCTTCCTTTACG
B1_bambia_26_Dla50	GAGGAGGGCAGCAAACGGaaTCCTTTCGTGAGAAGAACAGCCATC
B1_bambia_26_Dla50	CGGTGCGTCACAGTAGCACCTGATCtaGAAGAGTCTTCCTTTACG
B1_bambia_26_Dla50	GAGGAGGGCAGCAAACGGaaACCAGGCGATCCATTTACGGCTGCT
B1_bambia_26_Dla50	CAAAGTTCCAGCTGAAACCACAGAGtaGAAGAGTCTTCCTTTACG
B1_bambia_26_Dla50	GAGGAGGGCAGCAAACGGaaCCTCCGAATGGTTTGAGAGGACTCG
B1_bambia_26_Dla50	CCTTGTCTTGATACCCCGTGGCAAGtaGAAGAGTCTTCCTTTACG
B1_bambia_26_Dla50	GAGGAGGGCAGCAAACGGaaCCCAGGGATCTGTGATCTACACTGT
B1_bambia_26_Dla50	CATATGTCCTACATTTATTACTGCAtaGAAGAGTCTTCCTTTACG

Sequence name	Sequence
B2_gata2a_42_Dla100	CCTCGTAAATCCTCATCAaaTGTGGTTCGGCCCAGGCGGGAGAGC
B2_gata2a_42_Dla100	CTGTGGAGGGGATGCTGGGTTGTTCaaATCATCCAGTAAACCGCC
B2_gata2a_42_Dla100	CCTCGTAAATCCTCATCAaaGGTGAATGGGGGTCGGCGTGGGCAG

B2_gata2a_42_Dla100	CGGAATGGTGCGGGTGGCTGAATGTaaATCATCCAGTAAACCGCC
B2_gata2a_42_Dla100	CCTCGTAAATCCTCATCAaaGTGGCCCATGTGTGGCATGTGGCTT
B2_gata2a_42_Dla100	GTGTCCGGAATGGCTGAACGGTGGCaaATCATCCAGTAAACCGCC
B2_gata2a_42_Dla100	CCTCGTAAATCCTCATCAaaGTCTTGTCCTGCATGCACTTGGAGA
B2_gata2a_42_Dla100	AGGGCGCTGGCGCTGCCAAACGGAGaaATCATCCAGTAAACCGCC
B2_gata2a_42_Dla100	CCTCGTAAATCCTCATCAaaTCCTCTTGGACTTGCTGGACATCTT
B2_gata2a_42_Dla100	CCTCGAAACCCTCACCAGATCGTTTaaATCATCCAGTAAACCGCC
B2_gata2a_42_Dla100	CCTCGTAAATCCTCATCAaaCATGGTCAGTGGCCTGTTGACGTTG
B2_gata2a_42_Dla100	GTTGCGTGTTTGGATGCCCTCCTTCaaATCATCCAGTAAACCGCC
B2_gata2a_42_Dla100	CCTCGTAAATCCTCATCAaaCACACCGGGTCACCGTTCCCGTTGC
B2_gata2a_42_Dla100	AGTTTGTAGTAGAGCCCGCAGGCGTaaATCATCCAGTAAACCGCC
B2_gata2a_42_Dla100	CCTCGTAAATCCTCATCAaaAGTTCGCGCAGCAGGTGCCAGCTCG
B2_gata2a_42_Dla100	GCCACAGGGTGGTCGTGGTTGTCTGaaATCATCCAGTAAACCGCC
B2_gata2a_42_Dla100	CCTCGTAAATCCTCATCAaaGATAAGGGGTCTGTTCTGGCCGTTC
B2_gata2a_42_Dla100	CGCCGCAGACAGTCTGCGCTTTGGCaaATCATCCAGTAAACCGCC
B2_gata2a_42_Dla100	CCTCGTAAATCCTCATCAaaAGGTAGTGGCCCGTTCCATCCCGCC
B2_gata2a_42_Dla100	TTGTGGTACAGGCCGCACGCGTTGCaaATCATCCAGTAAACCGCC
B2_gata2a_42_Dla100	CCTCGTAAATCCTCATCAaaTCACACATTCACGCCCCTCTGAGCA
B2_gata2a_42_Dla100	ACAGCGGGGTGGAGGTGGCTCCACAaaATCATCCAGTAAACCGCC
B2_gata2a_42_Dla100	CCTCGTAAATCCTCATCAaaGAAGCTGGAAGCGGATCCGCTGAGG
B2_gata2a_42_Dla100	TCGCGTCTTGCTTTTACATTTAGGTaaATCATCCAGTAAACCGCC
B2_gata2a_42_Dla100	CCTCGTAAATCCTCATCAaaTAGTCGTGTGGCGCGGGCAGGGAGT
B2_gata2a_42_Dla100	GTACCGGGATGAAACAGGCCGCCGCaaATCATCCAGTAAACCGCC
B2_gata2a_42_Dla100	CCTCGTAAATCCTCATCAaaTGGAGGGTGTCTGTGCACTCATAGC
B2_gata2a_42_Dla100	TTGGGTAGGTCGGGATGGGATGATGaaATCATCCAGTAAACCGCC
B2_gata2a_42_Dla100	CCTCGTAAATCCTCATCAaaCATTTTCATGCCATCTGCGATGGAC
B2_gata2a_42_Dla100	GCTTCCCCGAAGAGGACTACATCCCaaATCATCCAGTAAACCGCC
B2_gata2a_42_Dla100	CCTCGTAAATCCTCATCAaaATTCTTGCAGTGGTGGATGTCGGAG
B2_gata2a_42_Dla100	TGGTACTTGATGGACTCCTTTTCGTaaATCATCCAGTAAACCGCC
B2_gata2a_42_Dla100	CCTCGTAAATCCTCATCAaaTGGAGTGGGTTGCGGATGTGAGGGA
B2_gata2a_42_Dla100	GGGGGAAATTATAAAGGGGATGCGGaaATCATCCAGTAAACCGCC
B2_gata2a_42_Dla100	CCTCGTAAATCCTCATCAaaGCACGGATAAGCGGCGCTGGCGGGG
B2_gata2a_42_Dla100	AACTGGAGCCGTGCTTGAACTGCTGaaATCATCCAGTAAACCGCC
B2_gata2a_42_Dla100	CCTCGTAAATCCTCATCAaaGCCCAGGCGTTGTGGTGGGCGGCGG
B2_gata2a_42_Dla100	AGGCCGGGCTTGCTGAAGTGGCTGAaaATCATCCAGTAAACCGCC
B2_gata2a_42_Dla100	CCTCGTAAATCCTCATCAaaGAATACCGGGACTGTGTATGAGGTG
B2_gata2a_42_Dla100	GCGCTGCCTTCCCGCTGTCCAGCCAaaATCATCCAGTAAACCGCC

107

Sequence name	Sequence
B3_szl_23_Dla100	GTCCCTGCCTCTATATCTttATCTTTACAAATTACATCTGAATAG
B3_szl_23_Dla100	ACATACAATACACTTATACATCTGAttCCACTCAACTTTAACCCG
B3_szl_23_Dla100	GTCCCTGCCTCTATATCTttCAACAGTTGGAGTGCATCTCAAGTC
B3_szl_23_Dla100	ATAATAATAATATTTCATCTCTATGttCCACTCAACTTTAACCCG
B3_szl_23_Dla100	GTCCCTGCCTCTATATCTttTGTGTGTGTGTTAAATCATATCTAG
B3_szl_23_Dla100	GATCAAAGAGCTGTGTGTGTGTGTGttCCACTCAACTTTAACCCG
B3_szl_23_Dla100	GTCCCTGCCTCTATATCTttAATGTGAGGTACAATACCATTTGCG
B3_szl_23_Dla100	ACAATATTGTAAGCAAAAATGAATAttCCACTCAACTTTAACCCG
B3_szl_23_Dla100	GTCCCTGCCTCTATATCTttTCTGCATCTGCAATTCGAACAAAGT
B3_szl_23_Dla100	AAAAACTTGAAATGTTTGTTTTGGTttCCACTCAACTTTAACCCG

B3_szl_23_Dla100	GTCCCTGCCTCTATATCTttCATAACCACACCCCTCTTATTTGTA
B3_szl_23_Dla100	TATATTCTTAGCCACACCCCTTTTAttCCACTCAACTTTAACCCG
B3_szl_23_Dla100	GTCCCTGCCTCTATATCTttCTCTTATTTGTAGCCACGCCCCTCT
B3_szl_23_Dla100	ACACCTGTCTTATCCATAACCACACttCCACTCAACTTTAACCCG
B3_szl_23_Dla100	GTCCCTGCCTCTATATCTttGGAAGTACGTCAACATACTGAAATA
B3_szl_23_Dla100	CCCCCTGCTTAATATTCAGTTTCAGttCCACTCAACTTTAACCCG
B3_szl_23_Dla100	GTCCCTGCCTCTATATCTttATAAGTTGCGCTTTCTCTCAGCACT
B3_szl_23_Dla100	TTTTTCTATATTTTTATAAATATATttCCACTCAACTTTAACCCG
B3_szl_23_Dla100	GTCCCTGCCTCTATATCTttCGATGTGCAAGTCTTTCTTCAGCCA
B3_szl_23_Dla100	AGTGTTTCCACTTGCGTGTTGCCGTttCCACTCAACTTTAACCCG
B3_szl_23_Dla100	GTCCCTGCCTCTATATCTttACCAGCACGCATTTTCCCGGTGATG
B3_szl_23_Dla100	GAACAGATTAGCGATCTCGATGGATttCCACTCAACTTTAACCCG
B3_szl_23_Dla100	GTCCCTGCCTCTATATCTttAGCGCATGCGCACAGCGGAGGTTGA
B3_szl_23_Dla100	TATAACTGCGCACGGCCGGGTCTGAttCCACTCAACTTTAACCCG
B3_szl_23_Dla100	GTCCCTGCCTCTATATCTttTGTCATAGGGCATTAGCGGCCCGCG
B3_szl_23_Dla100	GCATCCAGCGCTGCAGCAGACTAGCttCCACTCAACTTTAACCCG
B3_szl_23_Dla100	GTCCCTGCCTCTATATCTttAAACTCCGGCTCGGAAGACGGCACA
B3_szl_23_Dla100	GATCAGCTCCACAGCGCCCTCTACTttCCACTCAACTTTAACCCG
B3_szl_23_Dla100	GTCCCTGCCTCTATATCTttAAGTCGTTGAGGCAGAGCGAGTCCA
B3_szl_23_Dla100	CGACGCATGATCTTCACCTTCACCGttCCACTCAACTTTAACCCG
B3_szl_23_Dla100	GTCCCTGCCTCTATATCTttCTGATGGACAGCTCTGACATGCTGG
B3_szl_23_Dla100	GGGTTTTGAGTGACGGAGACTCCTGttCCACTCAACTTTAACCCG
B3_szl_23_Dla100	GTCCCTGCCTCTATATCTttGTGTTTGGGCAGCGGCGTCAGACAG
B3_szl_23_Dla100	AGGGAACTCTTTGGAGAAAGCACTGttCCACTCAACTTTAACCCG
B3_szl_23_Dla100	GTCCCTGCCTCTATATCTttAGTGATTCAGGCCAGGAGTGTCCGT
B3_szl_23_Dla100	TCCTGCTCCGGGAATCGCTCACAATttCCACTCAACTTTAACCCG
B3_szl_23_Dla100	GTCCCTGCCTCTATATCTttTTACTGCCAAACACACACTCCGGCA
B3_szl_23_Dla100	AGGCTAGAACGGGACTGCAGCTCTCttCCACTCAACTTTAACCCG
B3_szl_23_Dla100	GTCCCTGCCTCTATATCTttGGCGATGAGCGAGCAGACGAAGGCC
B3_szl_23_Dla100	CTGGATGAACCTGTCGAGGCATACAttCCACTCAACTTTAACCCG

**Sequence name**	**Sequence**
B1_spry4_55_Dla200	GAGGAGGGCAGCAAACGGaaGCAATGGGGACTCGGAATCCTTCAG
B1_spry4_55_Dla200	CTGCCACGAGGATACCTGGCGTCTTtaGAAGAGTCTTCCTTTACG
B1_spry4_55_Dla200	GAGGAGGGCAGCAAACGGaaTCAAACAACACAAGAAATAAAAGCT
B1_spry4_55_Dla200	CACGCAATCACCTCCCATGTTTGCAtaGAAGAGTCTTCCTTTACG
B1_spry4_55_Dla200	GAGGAGGGCAGCAAACGGaaAGAGATGAAGTCTGGTTATTTTGAT
B1_spry4_55_Dla200	GAACAAGCTCGTAAATGCACTGTGCtaGAAGAGTCTTCCTTTACG
B1_spry4_55_Dla200	GAGGAGGGCAGCAAACGGaaTGCACTTTCTGTCTTCCGGATCCGG
B1_spry4_55_Dla200	GACATGTGTTATCAGCTTGGAATTCtaGAAGAGTCTTCCTTTACG
B1_spry4_55_Dla200	GAGGAGGGCAGCAAACGGaaGAATGGGTGGTTTCTCAAAGAGAAA
B1_spry4_55_Dla200	GGGATTGCCAAAGTGGAGAACGCTTtaGAAGAGTCTTCCTTTACG
B1_spry4_55_Dla200	GAGGAGGGCAGCAAACGGaaTCTGTGTTTGCAAGGTCCTTTAGTA
B1_spry4_55_Dla200	AACAGAGATCTTTTGGAAATGCAGCtaGAAGAGTCTTCCTTTACG
B1_spry4_55_Dla200	GAGGAGGGCAGCAAACGGaaAAGAACTTCTTTGCAGTACGTGAAA
B1_spry4_55_Dla200	GCTAATACGTAGAACCATCGTGAGGtaGAAGAGTCTTCCTTTACG
B1_spry4_55_Dla200	GAGGAGGGCAGCAAACGGaaCTACTGATTTCAACACCTTAAGTAC
B1_spry4_55_Dla200	TCATTTGTGCCCTTCAGTCAAACATtaGAAGAGTCTTCCTTTACG

B1_spry4_55_Dla200	GAGGAGGGCAGCAAACGGaaATCATGAGGCTTGTTTTCTGGCTG
B1_spry4_55_Dla200	GATTTTGGGAGGAAGGTCCTGCAAAtaGAAGAGTCTTCCTTTACG
B1_spry4_55_Dla200	GAGGAGGGCAGCAAACGGaaCTGGGTGCTTTTGCATCGGCATCCT
B1_spry4_55_Dla200	GGCCTTGATTTCTGCCACCTTGCAGtaGAAGAGTCTTCCTTTACG
B1_spry4_55_Dla200	GAGGAGGGCAGCAAACGGaaGAAAGCTTGGCACAGCCAGTGGCAG
B1_spry4_55_Dla200	CGGCTGACGCCGTCGTAGCATTTCTtaGAAGAGTCTTCCTTTACG
B1_spry4_55_Dla200	GAGGAGGGCAGCAAACGGaaATGAGACGGCTGCCATGAAGGACCA
B1_spry4_55_Dla200	AATAGCACACCAGGCAGGGCAAAACtaGAAGAGTCTTCCTTTACG
B1_spry4_55_Dla200	GAGGAGGGCAGCAAACGGaaGCATGGTTTGTCCGCGCAGGAGCCT
B1_spry4_55_Dla200	TGCGCAACAGTTCGAGTGCGAACAGtaGAAGAGTCTTCCTTTACG
B1_spry4_55_Dla200	GAGGAGGGCAGCAAACGGaaACCCCCTTGACTAGACACATGCAAG
B1_spry4_55_Dla200	TCGTCCTCATCAGTGCAGTGGTAGAtaGAAGAGTCTTCCTTTACG
B1_spry4_55_Dla200	GAGGAGGGCAGCAAACGGaaACAGACACTCTTGGTTGCAAACCCA
B1_spry4_55_Dla200	CCGAGTCCACTAGGTTTTGTGCGGAtaGAAGAGTCTTCCTTTACG
B1_spry4_55_Dla200	GAGGAGGGCAGCAAACGGaaTTCGGTGCATCGGCATTTCCCGCAT
B1_spry4_55_Dla200	AGAAGGCAAGGTTCGGGGCAGCGTGtaGAAGAGTCTTCCTTTACG
B1_spry4_55_Dla200	GAGGAGGGCAGCAAACGGaaTCTTCAGGCAAGGCTGCCAGTGTCT
B1_spry4_55_Dla200	TCGCACAGCAGCACATGCTTTTTCTtaGAAGAGTCTTCCTTTACG
B1_spry4_55_Dla200	GAGGAGGGCAGCAAACGGaaGCTCCGCAGCTAGAGTCCTGCCGTG
B1_spry4_55_Dla200	TGTTCTTGGAGCTCAGTATCTTGGGtaGAAGAGTCTTCCTTTACG
B1_spry4_55_Dla200	GAGGAGGGCAGCAAACGGaaAGGAGTAGGAGCTGCGTGATCCAGC
B1_spry4_55_Dla200	ATTCCCGGTCGTATACGGATCCACAtaGAAGAGTCTTCCTTTACG
B1_spry4_55_Dla200	GAGGAGGGCAGCAAACGGaaCTGCTGATGGAGCTAGGCCGACCGC
B1_spry4_55_Dla200	CGCTGATCTGAAGACGTGCTACTGCtaGAAGAGTCTTCCTTTACG

110

**Sequence name**	**Sequence**
B2_dusp6_49_Dla100	CCTCGTAAATCCTCATCAaaGTGAAGTACAGTGGCTGGGTTGGGG
B2_dusp6_49_Dla100	TGAAAAACGTTGTGATTGGTTGGTGaaATCATCCAGTAAACCGCC
B2_dusp6_49_Dla100	CCTCGTAAATCCTCATCAaaGTCCTAACGTGCGCTCAAAGTCCAA
B2_dusp6_49_Dla100	GCACACGGTTATCACACGGACTCTTaaATCATCCAGTAAACCGCC
B2_dusp6_49_Dla100	CCTCGTAAATCCTCATCAaaGATGTTCGACTTCTTCATTTTGACA
B2_dusp6_49_Dla100	TTGACCCATGAAGTTAAAGTTGGGCaaATCATCCAGTAAACCGCC
B2_dusp6_49_Dla100	CCTCGTAAATCCTCATCAaaAGCTTCTGCATGAGGTACGCCACTG
B2_dusp6_49_Dla100	TCATAAGCATCGTTCATGGACAGGTaaATCATCCAGTAAACCGCC
B2_dusp6_49_Dla100	CCTCGTAAATCCTCATCAaaGGCAGTGAACAAGCACGCCACACTT
B2_dusp6_49_Dla100	CAGTGACAGAACGACTGATGCCTGCaaATCATCCAGTAAACCGCC
B2_dusp6_49_Dla100	CCTCGTAAATCCTCATCAaaGGCTTCAGGGAAAAACTGTGAGAGG
B2_dusp6_49_Dla100	TCCACGGGCCTCATCAATAAAGCTGaaATCATCCAGTAAACCGCC
B2_dusp6_49_Dla100	CCTCGTAAATCCTCATCAaaTGCTTGTACTTAAACTCCCCGGCAT
B2_dusp6_49_Dla100	TGGCTCCAGTGATCAGAGATGGGAAaaATCATCCAGTAAACCGCC
B2_dusp6_49_Dla100	CCTCGTAAATCCTCATCAaaCGTTCAAGATGTACTTGATGCCAAA
B2_dusp6_49_Dla100	CGAACATGTTGGGGAGATTAGGGGTaaATCATCCAGTAAACCGCC
B2_dusp6_49_Dla100	CCTCGTAAATCCTCATCAaaCTTAGCACAGCCCAGATACAGATGT
B2_dusp6_49_Dla100	CTCCAGGATATCCAGGTTTGTGGAGaaATCATCCAGTAAACCGCC
B2_dusp6_49_Dla100	CCTCGTAAATCCTCATCAaaGGGTTGGAAAGGGGGCTGCCATCTG
B2_dusp6_49_Dla100	AGGATCTCCACGGGGAATGAGGGCTaaATCATCCAGTAAACCGCC
B2_dusp6_49_Dla100	CCTCGTAAATCCTCATCAaaTATCCGACTCGATGTCCGAGGAGTC
B2_dusp6_49_Dla100	CAGTTGCACTGCTTGGGTCTCGGTCaaATCATCCAGTAAACCGCC
B2_dusp6_49_Dla100	CCTCGTAAATCCTCATCAaaGACCTGGGAGGTTGGGGAACTGCTG

B2_dusp6_49_Dla100	GCTGATTCTGAGCCCTCCGAGACCCaaATCATCCAGTAAACCGCC
B2_dusp6_49_Dla100	CCTCGTAAATCCTCATCAaaATTGCGGGAAAATCAGTTTGAAATT
B2_dusp6_49_Dla100	GAGGAACCGTCGAGGTTCGTCTCACaaATCATCCAGTAAACCGCC
B2_dusp6_49_Dla100	CCTCGTAAATCCTCATCAaaTGTAGCCCTCGTCCTTCATTCTCCT
B2_dusp6_49_Dla100	TGAAGCCACCCTCGAGATAGAAAGCaaATCATCCAGTAAACCGCC
B2_dusp6_49_Dla100	CCTCGTAAATCCTCATCAaaGATGTTTTCATTCCACTCGCGGCTG
B2_dusp6_49_Dla100	CAGTAAACCCAACACGGAGCCGCCGaaATCATCCAGTAAACCGCC
B2_dusp6_49_Dla100	CCTCGTAAATCCTCATCAaaTTGCATCTCCGCGCGAACCTTTCCC
B2_dusp6_49_Dla100	TCGTCGTACAACACGATCGTGTCCGaaATCATCCAGTAAACCGCC
B2_dusp6_49_Dla100	CCTCGTAAATCCTCATCAaaTGGGCAGGTTGCCTTTCTTGAGTCG
B2_dusp6_49_Dla100	CTTCCCCGTTAGAAAGCAGAGACTTaaATCATCCAGTAAACCGCC
B2_dusp6_49_Dla100	CCTCGTAAATCCTCATCAaaAATGGCCGTTTCGACGTGCGACGAC
B2_dusp6_49_Dla100	GAGCATGAGGCTCGGGATGGCCACGaaATCATCCAGTAAACCGCC
B2_dusp6_49_Dla100	CCTCGTAAATCCTCATCAaaACGAGCAAACAGTCTCTGCGCGTTT
B2_dusp6_49_Dla100	TACAGCTCTTGCGCTCGGCAGTCCAaaATCATCCAGTAAACCGCC
B2_dusp6_49_Dla100	CCTCGTAAATCCTCATCAaaTGCTTATGGCCATGACCGAATCGAT
B2_dusp6_49_Dla100	GCTGCTCCTTCAGCCACTCTACCGTaaATCATCCAGTAAACCGCC

**Sequence name**	**Sequence**
B3_il17rd_52_Dla100	GTCCCTGCCTCTATATCTttCTGAATCTACTTTTTCAGGAATGGT
B3_il17rd_52_Dla100	TGAGTTTGACTTCATTTAGCACCAAttCCACTCAACTTTAACCCG
B3_il17rd_52_Dla100	GTCCCTGCCTCTATATCTttTTCCTTCTCCAGCCAATCAGGCTCC
B3_il17rd_52_Dla100	TTTATTAGGCAGCGGCGGAGGCATGttCCACTCAACTTTAACCCG
B3_il17rd_52_Dla100	GTCCCTGCCTCTATATCTttGCAACATATAACGAGCGCCCCGATT
B3_il17rd_52_Dla100	GTGACGTACTGATGCATGTTATAAAttCCACTCAACTTTAACCCG
B3_il17rd_52_Dla100	GTCCCTGCCTCTATATCTttTCGGCGGCTGTGGTTCACGATCGGT
B3_il17rd_52_Dla100	TGCAGAAGTAGTTGCGTTTGCTAACttCCACTCAACTTTAACCCG
B3_il17rd_52_Dla100	GTCCCTGCCTCTATATCTttGAAGAGTTGCGGCAGCTGATCCATC
B3_il17rd_52_Dla100	ACTCAGTTGGCGCGAATGCAATCGGttCCACTCAACTTTAACCCG
B3_il17rd_52_Dla100	GTCCCTGCCTCTATATCTttGTCGGGACATCGGTTTCATGGGAAT
B3_il17rd_52_Dla100	TTAAACTTCGGCGCCAGGCTGAGTGttCCACTCAACTTTAACCCG
B3_il17rd_52_Dla100	GTCCCTGCCTCTATATCTttGGTCGGAGGATTTCTGGTGCACTTC
B3_il17rd_52_Dla100	CGAAATACACAGACATGAAGCGCGAttCCACTCAACTTTAACCCG
B3_il17rd_52_Dla100	GTCCCTGCCTCTATATCTttCACGATGAACAGATCTCTGCTGCTG
B3_il17rd_52_Dla100	GAGTTTCTCTGAGATGATGGCGGAGttCCACTCAACTTTAACCCG
B3_il17rd_52_Dla100	GTCCCTGCCTCTATATCTttTTTTTCTCTTTGGATGTGGCTTTGC
B3_il17rd_52_Dla100	CTGGAGTCGCTAGCGCTCGGCTCTCttCCACTCAACTTTAACCCG
B3_il17rd_52_Dla100	GTCCCTGCCTCTATATCTttTCAGGCCTTTGGAGCAGACGGTGAT
B3_il17rd_52_Dla100	TGCGGTGGCGTTTCTCCACGAAGTGttCCACTCAACTTTAACCCG
B3_il17rd_52_Dla100	GTCCCTGCCTCTATATCTttCAACCAGGACATCTGACCCTCCTTA
B3_il17rd_52_Dla100	GAAGTGGGCTTCGTCGATGCGCCGGttCCACTCAACTTTAACCCG
B3_il17rd_52_Dla100	GTCCCTGCCTCTATATCTttGAAACCTCGCAGCCGCAGAAGTCCT
B3_il17rd_52_Dla100	ATCTCCAGGTGCTCCCAGAGGTCCAttCCACTCAACTTTAACCCG
B3_il17rd_52_Dla100	GTCCCTGCCTCTATATCTttCGAGGTGTTTGGCTCCGTCTCTGCT
B3_il17rd_52_Dla100	GGAAGAAGGCGAAGCTCTGGATGACttCCACTCAACTTTAACCCG
B3_il17rd_52_Dla100	GTCCCTGCCTCTATATCTttCCAGGGTCTGTCTGCGCTCAGAGCC
B3_il17rd_52_Dla100	GTAACAGATGAAGATTTTGGGTCTGttCCACTCAACTTTAACCCG
B3_il17rd_52_Dla100	GTCCCTGCCTCTATATCTttTCGTCCAGGTGAGAGTAGATGTTTT
B3_il17rd_52_Dla100	GTCTGCGATGAAGACTCCGAGCTCTttCCACTCAACTTTAACCCG

B3_il17rd_52_Dla100	GTCCCTGCCTCTATATCTttAGAGTGTGGCGAAGGCTGACATGAT
B3_il17rd_52_Dla100	GCTGTTTCTTGCGGCACATGACGGTttCCACTCAACTTTAACCCG
B3_il17rd_52_Dla100	GTCCCTGCCTCTATATCTttGATCGGCCCGGCCCACGGGGAGTGA
B3_il17rd_52_Dla100	CAGCGGGACGGTGATGGCCATGGCAttCCACTCAACTTTAACCCG
B3_il17rd_52_Dla100	GTCCCTGCCTCTATATCTttCTGGTGTTGTTGCTGTCATCACGAA
B3_il17rd_52_Dla100	TGGCTGACGTGATACTGTGTCTGTCttCCACTCAACTTTAACCCG
B3_il17rd_52_Dla100	GTCCCTGCCTCTATATCTttCTTGTAAAACACAAGTAGTTTTGGG
B3_il17rd_52_Dla100	CGATTGCATACGTTCCTGGAGTGACttCCACTCAACTTTAACCCG
B3_il17rd_52_Dla100	GTCCCTGCCTCTATATCTttTCTGAACGGTCCTTCCTGTCTGAGT
B3_il17rd_52_Dla100	GTTTTGCTCAGGTTTGCAGCGCTTGttCCACTCAACTTTAACCCG

112

Sequence name	Sequence
B1_noto_16_Dla100	GAGGAGGGCAGCAAACGGaaTTCATCTATATTCAAACATTTTGAC
B1_noto_16_Dla100	TTGATTTTAAAAATATATGTCCTTTtaGAAGAGTCTTCCTTTACG
B1_noto_16_Dla100	GAGGAGGGCAGCAAACGGaaGGCGTGTGTAAGTGACGCCTTAAAT
B1_noto_16_Dla100	AAACAAAGATACCCTTATAAGAGCAtaGAAGAGTCTTCCTTTACG
B1_noto_16_Dla100	GAGGAGGGCAGCAAACGGaaTAGAGAGGAGTAACAATCTGGGGAA
B1_noto_16_Dla100	TTTCTATTAATTAAATATTTGTACAtaGAAGAGTCTTCCTTTACG
B1_noto_16_Dla100	GAGGAGGGCAGCAAACGGaaTGTGAGAAAGTCTCAGTCTTGCAGT
B1_noto_16_Dla100	CATGGAAAAGTATGTACAAAAACACtaGAAGAGTCTTCCTTTACG
B1_noto_16_Dla100	GAGGAGGGCAGCAAACGGaaTCTTCTGTGAAATCCCTCTCCTCAT
B1_noto_16_Dla100	TCGTCAATGTCAATGTCTACATCAGtaGAAGAGTCTTCCTTTACG
B1_noto_16_Dla100	GAGGAGGGCAGCAAACGGaaGTGGAACGGTCAGTCCCAGTTTGGC
B1_noto_16_Dla100	CTCTGCCCTGGGATCCAGGGCTTTTtaGAAGAGTCTTCCTTTACG
B1_noto_16_Dla100	GAGGAGGGCAGCAAACGGaaCTTTCTCCATTTGATGCGCCTGTTC
B1_noto_16_Dla100	TTTGGCTTGTTGTTGCTCAAGACTCtaGAAGAGTCTTCCTTTACG
B1_noto_16_Dla100	GAGGAGGGCAGCAAACGGaaAGTTGGAGAGCAGATGCCAACAGAA
B1_noto_16_Dla100	AACCAGACTTTAACCTGAGCTTCAGtaGAAGAGTCTTCCTTTACG
B1_noto_16_Dla100	GAGGAGGGCAGCAAACGGaaGCGCGAACTCTTTCTCCAGTCTGGA
B1_noto_16_Dla100	GTTCAGATCCCACCATGTATTGCTGtaGAAGAGTCTTCCTTTACG
B1_noto_16_Dla100	GAGGAGGGCAGCAAACGGaaTCGCTTTGATTTCCCAGATTTGTGT
B1_noto_16_Dla100	CTGATCGTTGGTAAAACTTGTACGCtaGAAGAGTCTTCCTTTACG
B1_noto_16_Dla100	GAGGAGGGCAGCAAACGGaaAGTACTGCATCTTGTGCGTACACAG
B1_noto_16_Dla100	TAATGAGAATGAACTGCTGCTTTCGtaGAAGAGTCTTCCTTTACG
B1_noto_16_Dla100	GAGGAGGGCAGCAAACGGaaGCGGATAGCAGAACACGGGATAGCC
B1_noto_16_Dla100	CACGACATGTTGTTTGGAAGTTGTAtaGAAGAGTCTTCCTTTACG
B1_noto_16_Dla100	GAGGAGGGCAGCAAACGGaaCGCGAAGTGTGGCATCTGCGAGTAA
B1_noto_16_Dla100	CTGAGTTTGCATGATGCTTTGGCTGtaGAAGAGTCTTCCTTTACG
B1_noto_16_Dla100	GAGGAGGGCAGCAAACGGaaGTTTGGTTGGTTATGCTCCGGTACG
B1_noto_16_Dla100	AGCGGGAGCGCAGAGCTGGAGACAGtaGAAGAGTCTTCCTTTACG
B1_noto_16_Dla100	GAGGAGGGCAGCAAACGGaaCAGGCCTCGCGAGCAGAGCGTCTAT
B1_noto_16_Dla100	TTGTTCGTTCTCTCATCTCCGCCTGtaGAAGAGTCTTCCTTTACG
B1_noto_16_Dla100	GAGGAGGGCAGCAAACGGaaAGAAGAGGTGGCATAGTCCTGATGC
B1_noto_16_Dla100	GAATGATTTCCCGGTGCTCGGTTTTtaGAAGAGTCTTCCTTTACG

113

Sequence name	Sequence
B2_gsc_22_Dla20	CCTCGTAAATCCTCATCAaaCCTGTTTTCAGGCGACATTAAACTT
B2_gsc_22_Dla20	TTTTTAGATATTACTTTAATATTTGaaATCATCCAGTAAACCGCC
B2_gsc_22_Dla20	CCTCGTAAATCCTCATCAaaTGCCATCGTACATGTCTTCAGCTAC

B2_gsc_22_Dla20	ATTAATTAATGTCCGAATGTATCGTaaATCATCCAGTAAACCGCC
B2_gsc_22_Dla20	CCTCGTAAATCCTCATCAaaATCTTATCACGACAAGACTTTAAAA
B2_gsc_22_Dla20	CCATTCCAGAACATCAGATTTAGGTaaATCATCCAGTAAACCGCC
B2_gsc_22_Dla20	CCTCGTAAATCCTCATCAaaTATGGCTAGAATCCACGTTATTTG
B2_gsc_22_Dla20	TTTGCAGTATACACCAGGTAATATTaaATCATCCAGTAAACCGCC
B2_gsc_22_Dla20	CCTCGTAAATCCTCATCAaaTATCATTGTTAAAGGTAACATTTAC
B2_gsc_22_Dla20	GTAAATTTAAATTAATATAAATAACaaATCATCCAGTAAACCGCC
B2_gsc_22_Dla20	CCTCGTAAATCCTCATCAaaATTTACTCCAACTCACATACTTTAC
B2_gsc_22_Dla20	ACATCTGTGCAACAGCAAGACAACAaaATCATCCAGTAAACCGCC
B2_gsc_22_Dla20	CCTCGTAAATCCTCATCAaaGTGCAAGATTTCCCGTTCTCGTGTT
B2_gsc_22_Dla20	ATGTACAATAAAGTCCGAATTATATaaATCATCCAGTAAACCGCC
B2_gsc_22_Dla20	CCTCGTAAATCCTCATCAaaTTTTGCCCTCCTCAATTTTCTCTGA
B2_gsc_22_Dla20	TATATCAGCTGTCAGAATCCACGTCaaATCATCCAGTAAACCGCC
B2_gsc_22_Dla20	CCTCGTAAATCCTCATCAaaTGAGTTTTCTGATTCCTCTGACGAC
B2_gsc_22_Dla20	TGTTTTCGTGGATTTGTTCCATTTCaaATCATCCAGTAAACCGCC
B2_gsc_22_Dla20	CCTCGTAAATCCTCATCAaaCTGTTTTTGAACCAAACCTCTACCT
B2_gsc_22_Dla20	CTTTTCTGTCTTCTCCATTTTGCTCaaATCATCCAGTAAACCGCC
B2_gsc_22_Dla20	CCTCGTAAATCCTCATCAaaTGTAGCTGGTTGAGCAGCTGTAGCT
B2_gsc_22_Dla20	GTTCGGTGTCGTCTCTTGCGCCGACaaATCATCCAGTAAACCGCC
B2_gsc_22_Dla20	CCTCGTAAATCCTCATCAaaACGACATCATTTGATGTGGGACTGG
B2_gsc_22_Dla20	TTCTGGACAAGGTGCCCACGTTCATaaATCATCCAGTAAACCGCC
B2_gsc_22_Dla20	CCTCGTAAATCCTCATCAaaACCTGTATGAATACACGGACACTGT
B2_gsc_22_Dla20	AATAAGCACAGAGCCGGCGCTGTCAaaATCATCCAGTAAACCGCC
B2_gsc_22_Dla20	CCTCGTAAATCCTCATCAaaCAAGCTGGTCCAGTCGGCCCCTGGA
B2_gsc_22_Dla20	GAACCAAGGGTTGGTATTGCGCCACaaATCATCCAGTAAACCGCC
B2_gsc_22_Dla20	CCTCGTAAATCCTCATCAaaAGCCTATCCTTCCATTCACCGATTG
B2_gsc_22_Dla20	GAAGTTGTCCATAGTAGTAGTTGTTaaATCATCCAGTAAACCGCC
B2_gsc_22_Dla20	CCTCGTAAATCCTCATCAaaATACAGTCCATTAAAATCGCCAGCT
B2_gsc_22_Dla20	GTTCGGCGCTGGAGGTCCTGTGTGTaaATCATCCAGTAAACCGCC
B2_gsc_22_Dla20	CCTCGTAAATCCTCATCAaaAACACAACCGGGGCATTCCGGTGGA
B2_gsc_22_Dla20	GTGTACAAGGATTCCGTCAAGTTGGaaATCATCCAGTAAACCGCC
B2_gsc_22_Dla20	CCTCGTAAATCCTCATCAaaCGGCCAAGATGCTGTCGATACTAAA
B2_gsc_22_Dla20	GAACCGAGTCTTTGCAGCTGGGTCTaaATCATCCAGTAAACCGCC
B2_gsc_22_Dla20	CCTCGTAAATCCTCATCAaaGTGTGAGATTTGTTGCCAACGGTAA
B2_gsc_22_Dla20	CCCAGCGGGCATCACAAGCGAAAAGaaATCATCCAGTAAACCGCC
B2_gsc_22_Dla20	CCTCGTAAATCCTCATCAaaACGAGTGTCCTTCATAGTGACAAAA
B2_gsc_22_Dla20	CGTGTTATTTTAGTCCTTTTAAAAAaaATCATCCAGTAAACCGCC

114

**Sequence name**	**Sequence**
B3_lft1_22_Dla100	GTCCCTGCCTCTATATCTttCTACAAATCAATGGCATCATATACA
B3_lft1_22_Dla100	CCGTGCTATATGCTCAAAATAAAACttCCACTCAACTTTAACCCG
B3_lft1_22_Dla100	GTCCCTGCCTCTATATCTttTCTATTTACAAGTCTATACAAAGTG
B3_lft1_22_Dla100	GCAAAAATAAACGCATATCAGATTAttCCACTCAACTTTAACCCG
B3_lft1_22_Dla100	GTCCCTGCCTCTATATCTttAACACCCATTCTAAATCTTATAAGT
B3_lft1_22_Dla100	GAGGTATCTAGTAAGGGTTAAATTGttCCACTCAACTTTAACCCG
B3_lft1_22_Dla100	GTCCCTGCCTCTATATCTttTGGTCGATACAAACAGGCTATTTAT
B3_lft1_22_Dla100	GGTAGTATAGTGCGTCATGAAGATAttCCACTCAACTTTAACCCG
B3_lft1_22_Dla100	GTCCCTGCCTCTATATCTttCATTTTTCCACAATCATGTTTGGGA
B3_lft1_22_Dla100	ACTGAAATATTGTCCATTGCGCATCttCCACTCAACTTTAACCCG

B3_lft1_22_Dla100	GTCCCTGCCTCTATATCTttGCGCGCTCTCGACGACCGCGCATTT
B3_lft1_22_Dla100	TTTTTACTAGATACATCATCGGTAGttCCACTCAACTTTAACCCG
B3_lft1_22_Dla100	GTCCCTGCCTCTATATCTttCTGCCGGCAGCCGCCTTTACACCTG
B3_lft1_22_Dla100	CTCTCCGTAGCCGTAGTTGCGCTTTttCCACTCAACTTTAACCCG
B3_lft1_22_Dla100	GTCCCTGCCTCTATATCTttCAGTACTGTGTCCAAGTCAGAGCTC
B3_lft1_22_Dla100	GCCTGGTAACCGGACGGCTCGATGAttCCACTCAACTTTAACCCG
B3_lft1_22_Dla100	GTCCCTGCCTCTATATCTttACATTTCACGGTCTTTGTTGTTTTC
B3_lft1_22_Dla100	AATTGATGAAGTACTGTTCCCTGCAttCCACTCAACTTTAACCCG
B3_lft1_22_Dla100	GTCCCTGCCTCTATATCTttGTTGAGTGTGTAAAGCACCAGCTCT
B3_lft1_22_Dla100	GTCTCCACTAGACCCAAACTCCTCTttCCACTCAACTTTAACCCG
B3_lft1_22_Dla100	GTCCCTGCCTCTATATCTttTGAGTTGTGAAGTGGACACACTTGG
B3_lft1_22_Dla100	TTTCCCAGAGTGTTGTCGTCTGGGTttCCACTCAACTTTAACCCG
B3_lft1_22_Dla100	GTCCCTGCCTCTATATCTttCGCCCTCGATCCACACCTCAAGGTG
B3_lft1_22_Dla100	TCTCTGCCGCGTAACTGCCGGGTCTttCCACTCAACTTTAACCCG
B3_lft1_22_Dla100	GTCCCTGCCTCTATATCTttATATTGCACCGCCTGGGTGACATCA
B3_lft1_22_Dla100	GGGCATCTCCATTCGGCTCCTAGACttCCACTCAACTTTAACCCG
B3_lft1_22_Dla100	GTCCCTGCCTCTATATCTttATCAACCTGGAATCCACTAGTGAAG
B3_lft1_22_Dla100	CTCTTCCAGCCAGTTTCGTGAATGGttCCACTCAACTTTAACCCG
B3_lft1_22_Dla100	GTCCCTGCCTCTATATCTttCCCAGTAGATGCTCACTCGTGCGTT
B3_lft1_22_Dla100	GGTTTGACCCGTCTTTCTGAGGTTCttCCACTCAACTTTAACCCG
B3_lft1_22_Dla100	GTCCCTGCCTCTATATCTttTATGGAGCGCTTGTGTGGGGCCTTC
B3_lft1_22_Dla100	GACCGGTCTGTGGCCCTTTCTCTCCttCCACTCAACTTTAACCCG
B3_lft1_22_Dla100	GTCCCTGCCTCTATATCTttTCACTGTTCTCGGGGATTCTTGATG
B3_lft1_22_Dla100	TAGAGCTTCAGTTCTGCCATGGTCAttCCACTCAACTTTAACCCG
B3_lft1_22_Dla100	GTCCCTGCCTCTATATCTttTGTCAGAATATACGAATTCACCAGA
B3_lft1_22_Dla100	TTTCAAAGACCACACGCTGACGTGTttCCACTCAACTTTAACCCG
B3_lft1_22_Dla100	GTCCCTGCCTCTATATCTttGATGCCGGCCAAACTGGGAAGCGAG
B3_lft1_22_Dla100	GTCTGCATTTCCAGGAATTCCCCTCttCCACTCAACTTTAACCCG
B3_lft1_22_Dla100	GTCCCTGCCTCTATATCTttATGGAGATATATTTGTTCTTTACGT
B3_lft1_22_Dla100	CGTTTTCTTGAGTGGTGAAGTTTCAttCCACTCAACTTTAACCCG

Supplementary Information 1: Construct sequences. All constructs used here are in the ampicillin-resistant pCS2+ vector backbone and can be linearized for mRNA synthesis using NotI.

Key: **Myr**, **linker**, **LOV**, **putatitve kinase domain**, **HA tag**

bOpto-FGF (Addgene #232639)

atggggagtagcaagagcaagcctaaggaccccagccagcgcggtggaggaggttctggaggcggtggaagtggtggcggaggtagcctggccaaaatgcacagctctgccaagaaaagcgacttcaacagccagctggccgtccacaagctggccaagagcatcccccctgcgcagacaggtaacagtgtctgtggactccagctcatctatgcattcgggtgggatgttggtccgtccatcccgtctgtcctccagtggctccccaatgctctcaggggtctccgaatacgagcttccccaggacccacgctgggaggtgcaacgagacaggctggttctcgggaaacctcttggcgaaggctgctttggacaggtgatgatggccgaggcgatggggatggataaagaaaaacccaatagaatcaccaaagtggccgtcaagatgctcaaatcggatgccacggagaaagacctgtcagacctgatctctgagatggagatgatgaagattattggcaaacacaagaacatcatcaacctgctgggagcctgcacacaagacggtccgctgtacgtcatcgtggagtttgctgctaaagggaacctgcgggagtatctgcgcgtacggcgtccaccagggatggagtactgttataaccctgaccaggtgccagtggagaacatgtccattaaagacctggtgtcctgcgcatatcaggtggcccgaggaatggagtatctcgcatccaagaagtgtattcatcgagacctggctgctcggaatgtgctggtgacggaagataacgttatgaagatcgcagactttggcctggccagagacatccatcatattgattactacaagaagaccaccaatggtcgtttgccggtgaaatggatggctcccgaagctctgtttgaccgcatatacacccatcaaagtgacgtctggtcttttggggtgctgctgtgggagatcttcactctggggggctctccgtaccccggcgtccccgtcgaggagctctttaagctgctgaaggaaggacaccgcatggaccgaccctccacatgcacacatgagctgtatatgatgatgagggattgttggcacgccgtcccgtctcagagacccacttttaaacagctggtggaggatctggaccgcacccctctccatgacgtccaatcaggagtatctggacctgtccgtatctctggaccagttttctccaaacttcccggacactcgcagctccacctgctcctcaggtgaagactcagtgttttctcatgacgccggagccgatgagccctgtttgcccaaattcccaccccatcccaaccgaggagtggcctttaaaaagcgcggtggaggaggttctggaggcggtggaagtggtggcggaggtagccctgactacagtctcgtgaaggctctgcaaatggcacaacagaattttgtcattacagacgcctccctcccagacaaccctatcgtctacgccagtagagggtttctgacactgacaggctattctctcgaccagatcctgggcaggaactgcaggtttctgcaagggccagaaacagacccaagagctgtggataagatcaggaatgccatcaccaaaggcgttgataccagtgtctgtctgctgaattatagacaggatggcacaaccttctggaatctcttcttcgtggctggactcagagattctaagggcaatattgtcaactacgtcggagtgcagtcaaaggtgagcgaagattatgccaagctgctggtcaacgagcagaacattgagtacaaaggtgtgcgcaccagtaacatgctgcgcagaaagcccgggtctagttatccgtacgacgtaccagactacgcataa

bOpto-Bmpr1aa (Addgene # 207614):

atggggagtagcaagagcaagcctaaggaccccagccagcgcggtggaggaggttctggaggcggtggaagtggtggcggaggtagctacaggtataagtggcagacagagaggcagcgctaccacagagacctggagcaagacgaggcctttatcccagcaggagaatccctgaaagacctcatcaaccagtctcagacctcaggcagtggctctggactccctctgctggtgcagcgcactatagcaaagcagatccagacagtgcgaatgatcggaaaaggacgatatggagaagtgtggcttggtcggtggagaggagagaaggtagcagtgaaggtgttctttacccgagaggaggccagctggttcagagagacagagatctatcaaaccgtgctcatgagacatgagaacatactcggctttatcgctgctgatataaatggcacaggagcctctacgcagctgtacctgatcacagactaccatgagaatggctctctgtatgactatctgaagttcacgactttggacacacaggctctactcagactggccttctctgcagcctgtggcctgtgtcacctgcatacggagatctacggcacgcagggaaaaccagcgatcgctcacagagacctgaagagcaagaacattctcatcaagaaaaacggcacctgctgcatcgctgacctcggccttgctgtgaaattcaacagtgacacaaatgaagtggacctcccattaagcacacgtatgggaaccaggcgctacatggctccagaagtgttggacgagactctgaataagaatcatttccaggcctacatcatggcagacatctacagctatgggctggttatttgggaaatggccagacgctgtgtcactggagggattgttgaggagtatcagctgccatattatgagatggtgccttcagacccatcttatgaagacatgttggaggttgtttgtgtcaaaggactgcggcccaccgtatccaacagatggaacagtgatgagtgcttaagggccatgctaaagctgatgtctgaatgctgggcccacaatcctgcatcacgcttaaccatcctacgagtcaagaagactttagccaaaatggtggaatctcaagacattaaaatcggtggaggaggttctggaggcggtggaagtggtggcggaggtagccctgactacagtctcgtgaaggctctgcaaatggcacaacagaattttgtcattacagacgcctccctcccagacaaccctatcgtctacgccagtagagggtttctgacactgacaggctattctctcgaccagatcctgggcaggaactgcaggtttctgcaagggccagaaacagacccaagagctgtggataagatcaggaatgccatcaccaaaggcgttgataccagtgtctgtctgctgaattatagacaggatggcacaaccttctggaatctcttcttcgtggctggactcagagattctaagggcaatattgtcaactacgtcggagtgcagtcaaaggtgagcgaagattatgccaagctgctggtcaacgagcagaacattgagtacaaaggtgtgcgcaccagtaacatgctgcgcagaaagcccgggtctagttatccgtacgacgtaccagactacgcataa

bOpto-Acvr1l (Addgene # 207615):

atggggagtagcaagagcaagcctaaggaccccagccagcgcggtggaggaggttctggaggcggtggaagtggtggcggaggtag
ccgtcgactccatcatgggcgtctggagagactgcacgagtttgacactgaacagggggccatcgatgggcttatcgcgtctaatgtcgga
gacagcacacttgcggatctgatggatcactcctgcacttcaggcagtggttcaggactgcccttcctggttcagagaacggttgcgcggca
gatcagcctggtggagtgtgttggtaaaggacggtacggtgaagtgtggagaggtcaatggcaaggagaaaatgtagccgtgaagatcttt
tcctctagagatgagaagtcatggtttcgagaaacagaaatttacaacactgttctgctacgacatgaaaatatattaggcttcatggcttctga
catgacctcccgaaactctagcactcagctgtggctgatcacacactatcacgagaatggctctctgtatgactacctgcagcgtgtggctgt
ggagatggcagatggactgcacatggcggcttcgattgccagcgggctggtgcacctgcacacggagatctttggcacggagggcaaac
cggccatcgctcacagagacctgaagagcaagaacatccttgtgaagaaagatttgcagtgctgcatcgctgacctgggtctggcagtaac
acacacgcagtctgataatcagcttgatgtgggaaataatcctaaagtgggaaccaaacgctacatggcaccggaggttctagatgagacc
attcagacggactgttttgacgcctataagagggtggatatctgggcctttgggttggtgctgtgggagatcgcacgcagaaccatcagcaat
ggaattgtagaggaatacaagccgcctttctatgacctggttcctaatgatcccagctttgacgacatgaggaaagtggtttgtgtggagcag
caaaggccattcattcccaaccgctggttttcagatcctaccctgtctgctctggtgaagctgatgaaagagtgctggtaccagaacccctcg
gctcgtctcactgccctgcgcatcaaaaagactctggataaaatccacagttcactggagaagggcaaaaccgactgcggtggaggaggtt
ctggaggcggtggaagtggtggcggaggtagccctgactacagtctcgtgaaggctctgcaaatggcacaacagaattttgtcattacaga
cgcctccctcccagacaaccctatcgtctacgccagtagagggtttctgacactgacaggctattctctcgaccagatcctgggcaggaact
gcaggtttctgcaagggccagaaacagacccaagagctgtggataagatcaggaatgccatcaccaaaggcgttgataccagtgtctgtct
gctgaattatagacaggatggcacaaccttctggaatctcttcttcgtggctggactcagagattctaagggcaatattgtcaactacgtcgga
gtgcagtcaaaggtgagcgaagattatgccaagctgctggtcaacgagcagaacattgagtacaaaggtgtgcgcaccagtaacatgctg
cgcagaaagcccgggtctagttatccgtacgacgtaccagactacgcataa

bOpto-Bmpr2a (Addgene # 207616):

atggggagtagcaagagcaagcctaaggaccccagccagcgcggtggaggaggttctggaggcggtggaagtggtggcggaggtagccgcatgctaagaggacgtggtaaacactcactgcatacttttaaatatactggagacggcactttctcctccttctttggatctggacaacctcacactgcaggagctgattggccggggccggtacggtacagtgtatcgtgcttcgctggatgaccgatctgtagcagtgaaagttttcatttctgctaaccgccagcagttcactaatgagcgtatgatttatcgcctcctcctggatcacgagaacatagcgcgcttcctagagagcgaggagcgcgtcggcacagaaggtcggacagagtttctccttctgctggagttttatcctcatggctctctgtgcacgtatctgagcggccggactgtggactggttgagctgctgccgtttggctctgtctgtgaccagagggttggcgtacctacatacagagatacagcgaggggatgtgtataaaccggctgtctcccaccgggatctgaacagcaggaatgtgctggttaagacggacggctcgtgtgtgatcagtgactttggactgtccatgattctgataggaaagaggccgcctggtcatggagaagaggacaacagtgccatcagtgaggtaggtacagtgcggtacatggctccagaagtgctggaaggggctgtgaatctgagggattgtgagtctgcgctgaaacaagtggatgtttatgcactgggtctggtttactgggagaccttcatgcgctgtgcagatcttttcccaggtgaaacagtgccagcttttcagttggcattccaggcagaggtgggcaatcatcctaccatagaggacgtgcaggcgcttgtgtccagagaaaaagaaagacccaaattcccagaagcctggaaagagaatagcctgacggtgcattctctgaaagagacgatggaagactgctgggatcaggacgcagaagcccgactgacagctcagtgtgcggaggagcgactcgccgaactgctcctcatctgggacagagaaaaatcagccagtcctgctcttaaccacagcactgcactgcagacacctaaagtaggttctgtccttgaacactcatccacaaacactgaagaccatgaaggtgctgataaaccactccacaatgacacctcagtggagcaaacctcagcaggaggaactaatcccgcagagaagaaaaagaactgcatcaactatgagtggcagcaggcccaatcacgacagtctggcacagaaagctcctccgctactcctgtctcagagtcctgcaatgccacgcatcctccaccagtggccaccaatctctgtgctcagctgacacgagaagacctggagataccaaaactcgacccgagcgaagtccagaggaacatgagagagagctcggacgagagccttatggaacattcacagaaacagttctgctctcctgaaacgtcagcctcccacggcccgttttatcctctcatgaagatggtttctgaggtttcggggtcacaaggatccagtaggcatggggacacccctataaccatcttacctaagcagcagaacgtccctaagagacccagtagcctcagtctccacgtaaagcctgggaaaacatccacctcgacgtcttcatcgctgcggatgaagttcggaaagcttggaaagtcaaatctgaagaaggttgagatgggtgtggctaaaagcagtgtggtgaatgcgacgcatgaagcccggctgattacagttgccaacaacgatgcggcggcagcaatgaataaatatgcaacagaaccagcagccgagtcagcaggaagttcagccaacgaggacttgaccttcggcctcttaaacaccagtcccgatgagcaggagcctctgctgagaagagaggcgcatcctgacaatgcaaacaacaacaacagcaataacaacaatggtgagggagatggtgatggagagacagagggtggaggagagggaggagagaacaatgagagtgtcggtccgacaggggatgcttcgtcgtcttctacggtggagccggttgtgctgcctggcccgtgttctgcttcaaccgccattcctccacaggcccaaagccagacacagacacacggagaggctctgctcagacagaaccgagtgcgcagaccagagagacccaactctctggatctgtccatcacaacactgccattactaggaggcaggtctgtcggcgatgggacagaggggtcaggggataaaatcaagaagcgggtaaagacgccttacgcacttaagaagtggcgtcctgccagctgggttatcaccactgacacactggatgccgaagtcaacaacaacagtcgtcacggaggaggcctcgggcagaatcagaatcaggctgggaccagcagacctaaatcagcttcggctgtctatctaggcagtcgaggaggatctcgcttctcagatcctaatgactgtgactttggtggaggaggttctggaggcggtggaagtggtggcggaggtagccctgactacagtctcgtgaaggctctgcaaatggcacaacagaattttgtcattacagacgcctccctcccagacaaccctatcgtctacgccagtagagggtttctgacactga

caggctattctctcgaccagatcctgggcaggaactgcaggtttctgcaagggccagaaacagacccaagagctgtggataagatcagga
atgccatcaccaaaggcgttgataccagtgtctgtctgctgaattatagacaggatggcacaaccttctggaatctcttcttcgtggctggactc
agagattctaagggcaatattgtcaactacgtcggagtgcagtcaaaggtgagcgaagattatgccaagctgctggtcaacgagcagaaca
ttgagtacaaaggtgtgcgcaccagtaacatgctgcgcagaaagcccgggtctagttatccgtacgacgtaccagactacgcataa

bOpto-2A-Nodal (Addgene #232640)

Key: **Myr**, **linker**, **Acvr1b**, **LOV**, **HA tag**, **P2A**, **Acvr2b**, **FLAG tag**

atggggagtagcaagagcaagcctaaggaccccagccagcgcggtggaggaggttctggaggcggtggaagtggtggcggaggtag
ccaccggcagcgactggatgttgaggatccatcctgtgatcacctgtacttggccaaagacaagaccttacaggatctcatcttcgatctgtc
cacctctggttcagggtctgggctgcccttgttcgttcagaggactgtggccaggacaattgtactgcaggagatcataggaaaaggtcgctt
tggggaagtgtggagggggagatggagaggaggtgatgtggctgtgaagatcttctcatccagggaggaacgttcctggttccgtgaagc
tgagatttaccaaaccatcatgctccgccatgaaaacatcttgggcttcattgctgctgataataaagacaatggcacatggacacagctgtg
gctagtgtcagactaccatgagcatggctcattatttgactatctcaaccactactccgtcacaatcgaggggatgatcaagttatcgctttcag
ccgccagcggtctggcacatctgcacatggagatcctgggcacacaggggaaaccaggcatcgcacatcgtgacctcaaatccaaaaac
atcttggtgaaaaagaatggtacttgcgccatagcagatctagggctcgctgtacggcacgagtccatcactgatactatagacattgcaccc
aatcagagggtgggcactaaaagatatatggccccagaggtactagatgaaacgatcaacatgaagcattttgattctttcaagtgtgctgat
atctatgctttgggactggtatattgggagattgcacgacggtgtaatgctggaggtatccatgaagattatcagctaccctactatgacctggt
gccgtccgacccctccatagaagagatgaggaaggtggtgtgtgaccagaggcttcggcctaatgtgcccaactggtggcagagctatga
ggcgctgagagtgatgggaaagatcatgcgggagtgttggtatgccaacggagcggcgcggcttacagctctgcggattaagaagactct
ttcgcaactcagcgtccaagaagacattaagggtggaggaggttctggaggcggtggaagtggtggcggaggtagccctgactacagtct
cgtgaaggctctgcaaatggcacaacagaattttgtcattacagacgcctccctcccagacaaccctatcgtctacgccagtagagggtttct
gacactgacaggctattctctcgaccagatcctgggcaggaactgcaggtttctgcaagggccagaaacagacccaagagctgtggataa
gatcaggaatgccatcaccaaaggcgttgataccagtgtctgtctgctgaattatagacaggatggcacaaccttctggaatctcttcttcgtg
gctggactcagagattctaagggcaatattgtcaactacgtcggagtgcagtcaaaggtgagcgaagattatgccaagctgctggtcaacg
agcagaacattgagtacaaaggtgtgcgcaccagtaacatgctgcgcagaaagcccgggtctagttatccgtacgacgtaccagactacg
caggaagcggagctacaaacttcagcctgctgaagcaggctggagacgtggaggagaaccctggacctatggggagtagcaagagcaa
gcctaaggaccccagccagcgcggtggaggaggttctggaggcggtggaagtggtggcggaggtagcaaacctccgtacggacatgtg
gacgtcaatgaggatccaggcccatctcctccatctcctctggtgggtctgaagcctctgcagctgctggaggttaaagctcgcggacgctt
cggctgcgtctggaaggctcagatgatcaatgaatatgtagctgtcaagattttccccattcaggataagctgtcgtggcagaacgagcggg
agatgttttccactccgggaatgaaacatgataacctgctgcgcttcatcgctgctgagaaacgcggatctaacctggagatggagttctggc
tcatcactgagtttcatgagcggggctctctgacggactatctgaaggggaacgcagtgagctgggctgatctgtgtgtgatagcggagag
catggcctgtggtctggcgtatctgcatgaagacgtgccgcgctccaaaggagaaggccccaaaccagccatcgcacacagagacttcaa
gagcaagaatgtgatgctgaagatggacctcaccgccgtcattggggattttgggctggcggtgcggtttgagccggggaaaccgccggg
agacacacatggccaggtgggcacgaggaggtacatggccccggaggttctggaaggagccataaacttccagcgggactcctttctgc
ggatagacatgtacgccatgggcctggtgctgtgggagctggtgtcacgctgcaaagctgctgatggtcctgtggacgagtacatgctgcc

gtttgaggaggagatcggtcagcacccgtcgctggaggatctgcaggatgctgtggtccataagaagctgcggccggcgtttaaggactgctggctcaagcattcaggtctgtgtcagatgtgcgagaccatggaggagtgctgggatcatgacgcagaggctcgtctgtcggccggctgtgtgcaggagcgcatctctcagatccgccgcgtcagcagctccacctcagactgcctgttctccatggtgacctcgctcaccaacgtggacctgccgcccaaagagtccagcatcggcggcggcggcagtggcggcggcggctctggcggcggcggctctcctgactacagtctcgtgaaggctctgcaaatggcacaacagaattttgtcattacagacgcctccctcccagacaaccctatcgtctacgccagtagagggtttctgacactgacaggctattctctcgaccagatcctgggcaggaactgcaggtttctgcaagggccagaaacagacccaagagctgtggataagatcaggaatgccatcaccaaaggcgttgataccagtgtctgtctgctgaattatagacaggatggcacaaccttctggaatctcttcttcgtggctggactcagagattctaagggcaatattgtcaactacgtcggagtgcagtcaaaggtgagcgaagattatgccaagctgctggtcaacgagcagaacattgagtacaaaggtgtgcgcaccagtaacatgctgcgcagaaagcccgggtctagtgactacaaggacgacgacgacaagtga

GFP (modified from (Lim et al., 2009))

atgagtaaaggagaagaacttttcactggagttgtcccaattcttgttgaattagatggtgatgttaatgggtacaaattttctgtcagtggagag
ggtgaaggtgatgcaacatacggaaaacttacccttaaatttatttgcactactggaaaactacctgttccatggccaacacttgtcactactct
cacttatggtgttcaatgcttttcaagatatccagatcatatgaagcggcacgacttcttcaagagcgccatgcctgagggatacgtgcagga
gaggaccatcttcttcaaggacgacgggaactacaagacacgtgctgaagtcaagtttgagggagacaccctcgtcaacaggatcgagctt
aagggaatcgatttcaaggaggacggaaacatcctcggccacaagttggaatacaactacaactcccacaacgtatacatcatggccgaca
agcaaaagaacggcatcaaagccaacttcaagacccgccacaacatcgaagacggcggcgtgcaactcgctgatcattatcaacaaaata
ctccaattggcgatgaccctgtccttttaccagacaaccattacctgtccacacaatctgccctttcgaaagatcccaacgaaaagagagacc
acatggtccttcttgagtttgtaacggctgctgggattacacatggcatggatgaactatacaaataa

nls-Kaede

Key: **Nuclear localization signal**, **Kaede**

atgcctaagaagaagagaaaggtgatgagtctgattaaaccagaaatgaagatcaagctgcttatggaaggcaatgtaaacgggcaccagtttgttattgagggagatggaaaaggccatccttttgagggaaaacagagtatggaccttgtagtcaaagaaggcgcacctctcccttttgcctacgatatcttgacaacagcattccattatggtaacagggtttttgctaaatacccagaccatataccagactacttcaagcagtcgtttcccaaagggttttcttgggagcgaagcctgatgttcgaggacgggggcgtttgcatcgctacaaatgacataacactgaaaggagacactttttttaacaaagttcgatttgatggcgtaaactttcccccaaatggtcctgttatgcagaagaagactctgaaatgggaggcatccactgagaaaatgtatttgcgtgatggagtgttgacgggcgatattaccatggctctgctgcttaaaggagatgtccattaccgatgtgacttcagaactacttacaaatctaggcaggagggtgtcaagttgccaggatatcactttgtcgatcactgcatcagcatattgaggcatgacaaagactacaacgaggttaagctgtatgagcatgctgttgcccattctggattgccggacaacgtcaagaagagacctgctgctaccaagaaagctggacaggccaagaagaagaaactggactag

References

Lim, S. M., Pereira, L., Wong, M. S., Hirst, C. E., Van Vranken, B. E., Pick, M., Trounson, A., Elefanty, A. G. and Stanley, E. G. (2009). Enforced expression of Mixl1 during mouse ES cell differentiation suppresses hematopoietic mesoderm and promotes endoderm formation. *Stem Cells* 27, 363-374.
